# Differential and directional body coupling analysis in Parkinsonian gait

**DOI:** 10.1371/journal.pone.0361959

**Published:** 2026-11-04

**Authors:** Arifa Zahir, Jaehong Yu, Jin-Sun Jun, Kiwon Park, Ryul Kim, Hyundoo Jeong

**Affiliations:** 1 Department of Biomedical and Robotics Engineering, Incheon National University, Incheon, Republic of Korea; 2 Department of Industrial and Management Engineering, Incheon National University, Incheon, Republic of Korea; 3 Department of Neurology, Kangnam Sacred Heart Hospital, Hallym University College of Medicine, Seoul, Republic of Korea; 4 Department of Neurology, Seoul Metropolitan Government–Seoul National University Boramae Medical Center, Seoul National University College of Medicine, Seoul, Republic of Korea; University Health Network, CANADA

## Abstract

Parkinson’s disease (PD) is a progressive neurodegenerative disorder primarily characterized by motor impairments and altered whole-body coordination. Objective and accessible approaches for quantifying inter-limb and cross-directional coupling during gait remain limited in clinical settings. Using a prospectively collected dataset of individuals with PD and healthy controls (HC), we propose a comprehensive analytical framework based on human pose estimation to extract time-series kinematic signals from key body landmarks. Coupling strengths between body segments were quantified to characterize whole-body coordination patterns. Statistical filtering identified discriminative body coupling factors that significantly differentiated PD from HC. The identified factors demonstrated clear and consistent alterations in coordination patterns across groups. Furthermore, multiple machine learning classifiers validated their discriminative capacity, achieving robust classification performance. Correlation analyses between the top 10 pre-specified directional body coupling factors and MDS-UPDRS III scores demonstrated modest but statistically significant associations in the full PD cohort (*n* = 121), with 8 of 10 factors surviving FDR correction (ρ=0.27–0.31). These associations suggest that directional body coupling metrics capture clinically relevant variation in motor impairment severity beyond binary PD versus HC discrimination and may serve as potential digital biomarkers of PD-related motor dysfunction. Subtype-stratified analyses were conducted only as exploratory supplementary analyses, and larger, more balanced subtype cohorts are required before subtype-specific conclusions can be drawn. Overall, body coupling metrics provide reliable biomechanical markers of PD-related gait impairment and support the clinical potential of this non-invasive and cost-effective pipeline for early diagnosis and longitudinal monitoring.

## Introduction

Parkinson’s disease (PD) is a chronic and progressive neurodegenerative disorder haracterized by both motor and non-motor symptoms. The cardinal motor features of PD include bradykinesia, resting tremor, rigidity, and postural instability, whereas non-motor manifestations may include cognitive impairment, sleep disturbances, autonomic dysfunction, mood disturbances, and fatigue [1–3]. As the disease advances, these symptoms contribute to reduced mobility, loss of independence, increased fall risk, and decreased quality of life [4]. Among the motor impairments associated with PD, gait abnormalities such as reduced step length, slower walking speed, irregular stride timing, reduced arm swing, and impaired inter-limb coordination are among the most clinically important functional deficits [5–7]. Consequently, gait analysis has emerged as an important approach for early diagnosis, disease monitoring, and the design of therapeutic or assistive strategies.

Several efforts have been made to comprehensively analyze distinctive gait patterns in PD. Beyond conventional spatiotemporal gait parameters, recent studies have emphasized the importance of body coupling factors, which describe the dynamic coordination between different body segments during walking [8,9]. Body coupling analysis can quantify how upper-limb, lower-limb, trunk, and pelvic movements are temporally and spatially synchronized, capturing inter-segmental relationships such as arm–leg coordination, trunk–pelvis interaction, and hip–knee coupling. These measures provide information beyond isolated gait variables because they reflect how the body moves as an integrated motor system. In PD, impaired automatic motor control, rigidity, reduced arm swing, and postural instability may disturb normal inter-segmental coordination, making body coupling analysis a useful approach for identifying disease-related gait abnormalities. Alterations in these coordination patterns, such as disrupted hip–knee coupling, abnormal thorax–pelvis rotation, and reduced arm–leg synchronization, have been reported in PD [7,10,11]. These deficits, often more sensitive than conventional spatiotemporal parameters, may provide clinically meaningful digital biomarkers for disease progression and therapeutic effects [12,13].

Advancements in objective motor assessments have supported progress in this area. Laboratory studies have shown reduced shoulder–hip coordination [7], impaired arm–leg phase relationships during backward walking [10], and increased asymmetry in bilateral arm swing [14]. Ecological and free-living studies have extended these findings, with wearable sensors predicting PD conversion [15], validating arm swing metrics in real-world environments [16], and demonstrating scalable staging with markerless pose estimation [17]. More recently, video-based gait analysis has gained momentum, leveraging 2D pose estimation and deep learning approaches. Smartphone-based pipelines have enabled detection of freezing of gait [18], interpretable models have stratified motor severity states [19], and multi-view fusion approaches have enhanced screening accuracy [20]. Additional work has classified tremor severity using consumer-grade video [21] and improved dataset quality via weakly supervised learning [22].

Despite these advancements, several limitations in prior studies constrain their interpretability and clinical utility. Some investigations have focused only on a narrow subset of body segments, often analyzing arm movements in isolation while overlooking whole-body interactions such as upper–upper, lower–lower, and upper–lower limb coordination, which may limit a comprehensive understanding of global gait dynamics [23,24]. Moreover, such isolated analyses can reduce the ability to capture holistic gait characteristics, and failing to model inter-limb connections may reduce sensitivity to subtle motor impairments [25,26]. Other studies have exhibited directional biases, frequently restricting analyses to single-plane or homologous interactions [27,28]. For instance, single-axis analyses can obscure complex gait abnormalities that emerge in multi-planar movements [29]. Additionally, high-dimensional biomechanical signals are often simplified into coarse summary indices that collapse rich coordination data [30]. Overly aggregated metrics limit the ability to distinguish group-level differences [31,32], and preserving feature granularity is critical for detecting subtle but clinically meaningful variations in gait patterns [33].

To address these gaps, this study introduces an analytical pipeline that integrates 2D human pose estimation with statistical analysis methods for comprehensive whole-body coupling analysis by incorporating directional information. First, we collect gait data from PD patients and healthy control (HC) groups. We then extract the time-series coordinate changes of key body points using a human pose estimation method, MediaPipe. Second, based on the time-series coordinate changes of the key body points, we estimate the coupling strength among different points and retain the discriminative coupling factors that can distinguish PD patients from the HC group. Finally, through comprehensive performance assessment, we demonstrate that the identified discriminative body coupling factors reveal different coupling patterns in PD patients and yield improved low-dimensional visualization. Additionally, we show that the discriminative body coupling factors have the potential to separate PD and HC groups using diverse machine learning models.

The objectives of this research are to (i) identify discriminative body coupling factors differentiating PD patients from HC, (ii) assess their potential as digital biomarkers for early diagnosis and monitoring, and (iii) provide a comprehensive evaluation of upper–upper, lower–lower, and cross-limb interactions to enhance clinical interpretation of PD gait.

## Materials and methods

### Study population

In this prospective study, we recruited a total of 203 participants from the Department of Neurology at Inha University Hospital. This study was approved by the Institutional Review Board of Inha University Hospital (2022-09-037). The recruitment period for this study was from 12/12/2022–11/12/2023. All experiments involving human participants were performed in accordance with relevant guidelines and regulations. Written informed consent was obtained from all subjects and/or their legal guardians prior to participation, following a detailed explanation of the study procedures.

Patients with early-stage PD, defined as a disease duration of 3 years or less since diagnosis, were recruited from the Department of Neurology at Inha University Hospital during the study recruitment period. The diagnosis of PD was established by a movement disorder specialist (R.K.) according to the UK Parkinson’s Disease Society Brain Bank criteria [34]. Patients with other neurologic disorders, musculoskeletal conditions affecting gait, or severe cognitive impairment that could interfere with study participation were excluded. Healthy controls with a similar age distribution were recruited from family members of the patients or from volunteers willing to participate in the study. The same exclusion criteria were applied to the healthy controls.

Among these participants, 121 were patients with PD and 82 were healthy participants in the control group. There were 53 female and 68 male patients in the PD group, and 50 female and 32 male participants in the HC group. The average age of PD patients was 68.7±8.9 years, and that of the HC group was 65.3±9.8 years. The mean disease duration among the patients with PD was 1.4±1.2 years. The Hoehn and Yahr (HY) stage was 1.95±0.46, with a range of 1–3. The stage-wise distribution was HY 1, *n* = 13; HY 1.5, *n* = 14; HY 2, *n* = 73; HY 2.5, *n* = 15; and HY 3, *n* = 6. Overall, 115 of 121 patients with PD, corresponding to 95.0%, were classified as HY ≤2.5. The MDS-UPDRS-III score for PD patients ranged from 3 to 55, with a mean ± SD of 26.7±10.6 and a median of 25.5 as summarized in Table 1.

**Table 1:** Summary of study population characteristics. Values are presented as counts, ranges, mean ± SD, or median, unless otherwise specified.

Characteristic	PD Group (*n* = 121)	HC Group (*n* = 82)
Sex (Female / Male)	53 / 68	50 / 32
Age (years, mean ± SD)	68.7±8.9	65.3±9.8
Disease duration (years, mean ± SD)	1.4±1.2	–
Hoehn and Yahr stage (mean ± SD)	1.95±0.46	–
Hoehn and Yahr stage (range)	1–3	–
Hoehn and Yahr stage distribution, *n*	1: 13; 1.5: 14; 2: 73; 2.5: 15; 3: 6	–
MDS-UPDRS III score (range)	3–55	–
MDS-UPDRS III score (mean ± SD / median)	26.7±10.6 / 25.5	–
Motor subtype distribution, *n*	TD: 49; PIGD: 53; Indeterminate: 19	–

Motor subtypes were classified according to the MDS-UPDRS ratio-based criteria proposed by Stebbins et al. [35]. Patients were categorized as tremor-dominant (TD), postural instability and gait difficulty (PIGD), or Indeterminate. Among the 121 patients with PD, 49 were classified as TD, 53 as PIGD, and 19 as Indeterminate. Subtype-stratified analyses were performed as exploratory supplementary analyses to examine subtype-specific patterns of association between body coupling metrics and motor impairment severity.

With respect to comorbidities, we excluded patients with other neurologic disorders, musculoskeletal conditions affecting gait, and severe cognitive impairment that could interfere with study participation, thereby reducing the possibility of major confounding factors affecting our findings. Although metabolic and other systemic medical conditions were not systematically assessed in our cohort, such conditions were unlikely to have substantially influenced the primary study outcomes.

S1 Fig in S1 File shows the detailed distribution of Hoehn and Yahr stages.

### Data collection and preprocessing

To collect the gait patterns of participants, we used a smartphone to record their walking motions and applied a human pose estimation (HPE) open-source library to extract the coordinates of key body points. Prior validation studies have demonstrated that smartphone-based HPE approaches yield gait parameters comparable to those from laboratory-grade motion capture systems [36,37]. Since the smartphone-based HPE method is more affordable, portable, and easier to use, we adopted it for this study without compromising accuracy or quality.

In this study, we used a Samsung Galaxy S22 smartphone running Android OS, which has a video resolution of 2340×1080 pixels at 30 frames per second.

All recordings were performed in an indoor hospital corridor using a markerless setup with a plain white background to minimize visual interference during pose estimation. The smartphone was positioned laterally to the walking path, 3 meters from the participant, at a height of 0.8 meters, ensuring full-body visibility during gait cycles. Participants began each trial from quiet standing and took three to four forward steps before performing a 180° turn and returning to the starting position. Thus, the recorded walking sequences naturally included the gait-initiation phase, defined in this study as the transition from quiet standing to the first forward steps. Because the available walking distance was short in the hospital corridor, gait initiation and subsequent short-distance walking were not explicitly separated in the primary analysis. This protocol was selected to accommodate clinical constraints, including fall risk, fatigue susceptibility, and limited corridor space, while still allowing repeated walking sequences to be collected from each participant.

After recording, we extracted the two-dimensional coordinates (x- and y-axes) of specific body points using the MediaPipe library [38], chosen for its fast processing speed and low estimation error. All MediaPipe parameters were set to their default values, except for the model complexity parameter. The model complexity was set to 2, corresponding to the “Heavy” model configuration. For quality control, frames with unreliable body landmark detection, incomplete full-body visibility, or major keypoint tracking failure were excluded before subsequent coupling analysis. The human body was modeled as a graph *G* = (*V*, *E*), where nodes $v_{i}$ represent specific body landmarks (keypoints) (i.e., $v_{i}\in V$) and edges $e_{ij}\in E$ indicate anatomical connections between different keypoints on the body. This approach yielded time-series coordinate data for 18 body landmarks measured along two axes, producing 36 time-series signals per gait sequence, where changes along the x-axis corresponded to horizontal movements and changes along the y-axis indicated vertical movements in the recorded video. Abbreviations for these body landmarks, used throughout subsequent figures and tables to denote coupling factors, are defined in Table 2. Finally, the recorded bidirectional walking videos were segmented into single-direction walking sequences to build the final dataset, yielding 486 walking sequences for the PD group and 330 walking sequences for the HC group.

**Table 2:** Abbreviations for body landmarks used in coupling factor nomenclature. Each coupling factor name combines two landmark abbreviations (e.g., LELBx–RHEEy denotes the horizontal trajectory of the left elbow coupled with the vertical trajectory of the right heel). The prefix L/R denotes left/right side; for directional features, the suffix x/y denotes the horizontal/vertical axis, respectively.

Abbreviation	Body landmark
SHO	Shoulder
ELB	Elbow
WRI	Wrist
FIN	Finger
HIP	Hip
KNE	Knee
ANK	Ankle
HEE	Heel
TOE	Toe

### Identifying body coupling factors

We assumed that people with PD and HC would have noticeably different levels of coupling between different parts of the body when walking. Differences in the skeletal and motor systems that are necessary for human gait can be seen as changed coupling patterns. By continuous analysis, these patterns serve as a starting point for differentiating between gait profiles of PD and HC groups. To validate our assumption, we used a three-step process: (1) estimating paired body coupling matrices, (2) computing aggregated body coupling scores, and (3) identifying discriminative body coupling pairs through statistical analysis.

To quantify coordination between anatomically defined body points, we computed Spearman’s rank correlation coefficients between the time-series signals of all body keypoint pairs. Each gait sequence produced 36 time-series signals corresponding to 18 body points measured along two axes (i.e., horizontal and vertical directions). This resulted in a 36 by 36 correlation matrix per participant, yielding 630 unique coupled body pairs. We used the absolute Spearman correlation rather than the raw correlation values to focus on the strength of coupling rather than its direction. This is critical because both in-phase (positive correlation) and anti-phase (negative correlation) limb movements can represent strong and meaningful coordination patterns in human gait. By using absolute values, the analysis captured the magnitude of coupling strength between body parts, regardless of whether the movement relationship was aligned or opposite in phase.

To enhance interpretability and incorporate directional relevance, we aggregated the absolute Spearman correlations into a body coupling score. For each pair of body keypoints $\left(v_{i},v_{j}\right)$, we defined the body coupling score as:


$$\begin{array}{c} \text{Body Coupling Score}(v_{i},v_{j})=\sum_{\alpha \in \{x,y\}}\sum_{\beta \in \{x,y\}}\eta (\alpha ,\beta )\left|\rho ({𝐯}_{i}^{\alpha },{𝐯}_{j}^{\beta })\right|. \end{array}$$ (1)

where ${𝐯}_{i}^{\alpha }$ and ${𝐯}_{j}^{\beta }$ denote the time-series coordinate vectors of body keypoints $v_{i}$ and $v_{j}$ along the axis directions α and β (*x* or *y*), respectively. The term $\rho ({𝐯}_{i}^{\alpha },{𝐯}_{j}^{\beta })$ denotes the Spearman rank correlation coefficient between these two corresponding time-series coordinate vectors. The scaling coefficient η(α,β) adjusts the contribution of each axis combination in estimating body coupling strength. In this study, we hypothesize that same-axis couplings can play a pivotal role in gait patterns discriminating PD and HC groups, whereas cross-axis couplings have a lesser influence on the discriminative patterns. Based on this assumption, we empirically set the scaling coefficient as follows:


$$\begin{array}{c} \eta (\alpha ,\beta )=\left\{ \begin{array}{cc} 0.4, & \alpha =\beta \\ 0.1, & \alpha \neq \beta . \end{array}\right. \end{array}$$ (2)

Applying this formulation yielded a refined set of 153 aggregated body coupling scores per participant, reducing redundancy while preserving critical directional information. Based on the estimated body coupling scores, to identify the most discriminative coupled body pairs between PD and HC participants, we applied a two-step statistical filtering process. For each coupled body pair, Welch’s t-test compared the mean body coupling scores between PD and HC groups due to its robustness to unequal variances, a common characteristic of gait data in clinical populations. We retained only the body keypoint pairs with p-value less than 0.05 for further in-depth analysis.

Next, for the pairs that passed significance testing, we computed Cohen’s *d*, which serves as a quantitative and interpretable indicator of the effect size, thereby providing an efficient measure of the magnitude of group differences beyond the inferential evidence conveyed by the p-value alone.

Cohen’s *d* was calculated as:


$$\begin{array}{c} d=\frac{{\overset{―}{X}}_{\text{PD}}-{\overset{―}{X}}_{\text{HC}}}{s_{\text{pooled}}}, \end{array}$$ (3)

where ${\overset{―}{X}}_{\text{PD}}$ and ${\overset{―}{X}}_{\text{HC}}$ are the mean body coupling scores for the PD and HC groups, respectively, and *s*_pooled_ is the pooled standard deviation:


$$\begin{array}{c} s_{\text{pooled}}=\sqrt{\frac{(n_{\text{PD}}-1)s_{\text{PD}}^{2}+(n_{\text{HC}}-1)s_{\text{HC}}^{2}}{n_{\text{PD}}+n_{\text{HC}}-2}}. \end{array}$$ (4)

Here, $s_{\text{PD}}^{2}$ and $s_{\text{HC}}^{2}$ are the variances of the body coupling scores, and *n*_PD_ and *n*_HC_ are the sample sizes for the two groups. Finally, we retained only the discriminative coupling pairs with absolute Cohen’s *d* greater than 0.6 to ensure that the final set included clinically meaningful differences with moderate-to-strong effects. This stricter cutoff reduced noise, improved interpretability, and ensured the robustness of subsequent analyses.

This two-level filtering process was applied both to the aggregated body coupling scores (153 pairs) and to the original pairwise correlations (630 pairs), ensuring that both broad and detailed coordination patterns were considered. The final refined set of coupled body pairs formed a strong foundation for advanced machine learning classification and clinical interpretation, offering both statistical significance and clinically meaningful interpretation.

## Results and discussion

### Body coupling factors reveal directional differences between PD and healthy gait

Using our analysis framework, we identified 24 discriminative body coupling factors that exhibited significant differences in coupling strength between individuals with PD and HC, as determined by Welch’s t-test and a corresponding effect size of Cohen’s *d*. Interestingly, PD participants showed substantially stronger body coupling scores than those in the HC group, as shown in Fig 1A. This result supports the hypothesis that the strong coupling pattern can serve as one of the discriminative features for distinguishing between PD and HC groups.

**Fig 1:**
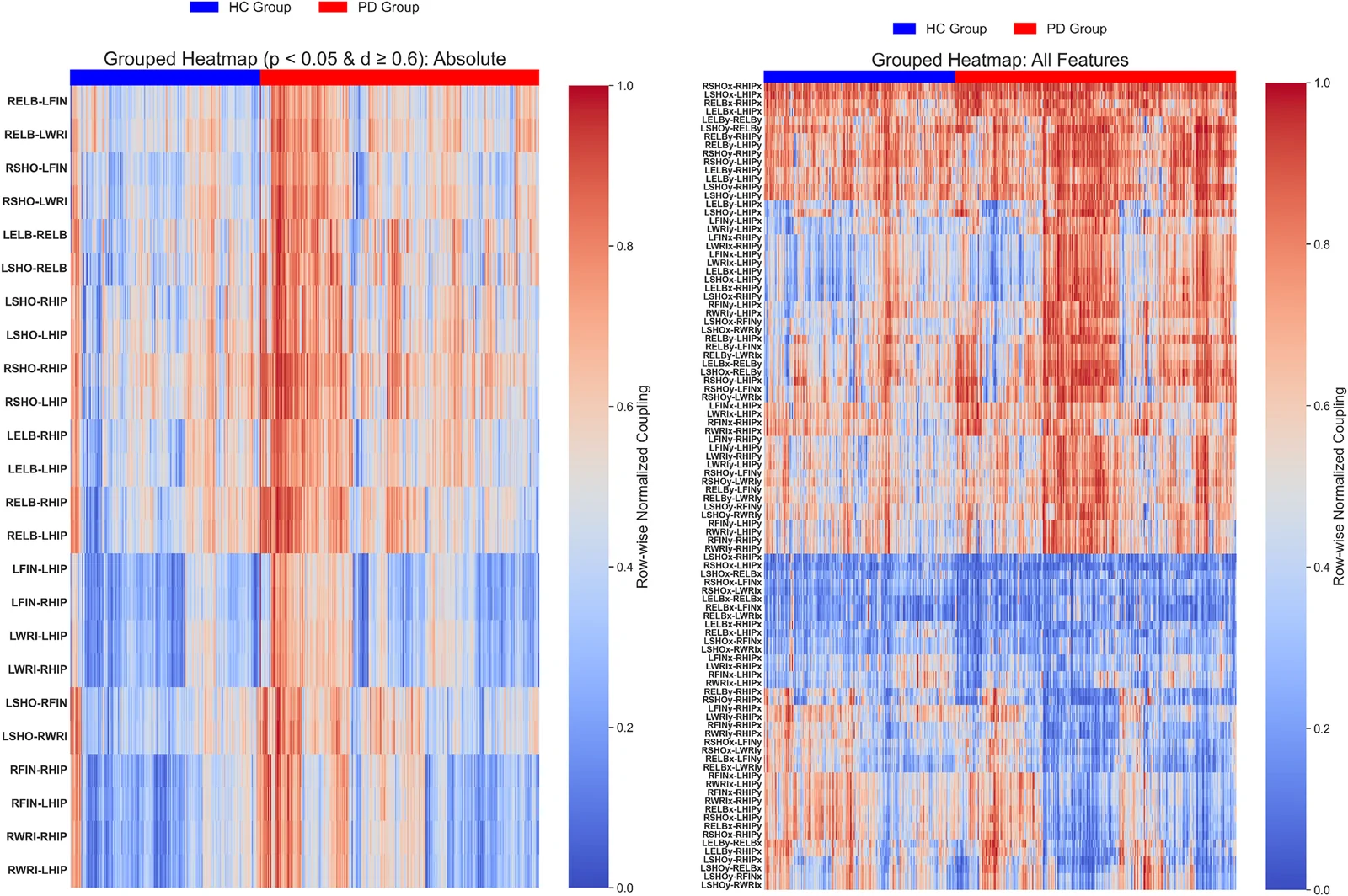
Grouped heatmaps illustrating the refinement process. (A) The 24 statistically significant unified body coupling factors. Color scale indicates group-level difference in mean coupling strength (PD minus HC); warmer colors indicate stronger coupling in PD. Row and column labels denote body landmark pairs. (B) The 96 directional coupling components highlighting axis-specific contributions. Each cell represents the signed difference in mean coupling strength for a specific axis combination (x or y) between landmark pairs. Color scale bar is shown on the right.

To conduct an in-depth anatomical analysis of the body coupling factors, we decomposed the identified discriminative factors according to their anatomical locations. The 24 discriminative coupling factors were classified into three anatomical groups: upper–upper limb, lower–lower limb, and upper–lower limb couplings. Note that upper–lower limb and lower–upper limb pairs differ only in order so that they can be regarded as the identical case. Among 24 discriminative body coupling factors, there are 8 discriminative factors corresponding to the upper–upper limb coupling and 16 corresponding to cross-limb couplings, as summarized in Table 3. PD participants typically exhibited stronger inter-limb coupling compared with HC participants, and these results suggest that altered inter-limb coordination may serve as a distinctive marker of PD-related gait impairments. Moreover, this symmetry indicates that PD-related gait dysfunction stems from disruptions in global coordination networks, rather than isolated segmental impairments, reinforcing the role of coordinated upper and lower limb dynamics in maintaining stable movement. S1 Table in S1 File reports the discriminative body coupling factors identified in this study.

**Table 3:** Anatomical decomposition of the 24 identified discriminative body coupling factors. The values indicate the number of discriminative factors. Upper–lower and lower–upper interactions denote the same limb-region pairing and are reported as a single combined cross-limb category.

	Upper limb	Lower limb
Upper limb	8	16
Lower limb	16	0

While these unified body coupling scores provided a detailed overview of inter-limb coordination, they lacked directional information regarding horizontal (x-axis) and vertical (y-axis) trajectories. To address this, since the coupling scores were derived by integrating all possible combinations of directional changes (i.e., each body coupling score incorporates four directional components), we decomposed the 24 discriminative couplings into 96 directional terms, enabling a more granular view of axis-specific contributions, as shown in Fig 1B. In contrast to the analysis results based on the body coupling scores, directional information indicates that while some directional body couplings still show strong coupling strength, others exhibit weaker coupling in the PD group relative to the HC group. These results motivate the hypothesis that body coupling analysis taking directional information into account can provide a more granular interpretation of discriminative gait patterns.

To gain a comprehensive view of inter-limb and their directional coordination, we examined the coupling strength among the 36 body pairs including their directional decompositions through the same analysis pipeline, thereby the analysis was expanded to the full set of 630 directional pairwise couplings. Based on absolute Spearman correlation coefficients of horizontal (x-axis) and vertical (y-axis) trajectories aggregated across gait cycles with statistical filtering based on a p-value cutoff of 0.05 and a Cohen’s *d* threshold of 0.6, we identified discriminative directional coupling factors. Again, we verified that there are discriminative coupling pairs with stronger coupling patterns that are consistent with the discriminative patterns identified from the body coupling scores, as shown in Fig 2. However, we also confirmed that some body pairs exhibit weaker coupling patterns that are not clearly captured through the integrated body coupling scores. This variability suggests the complexity of directional coupling mechanisms and suggests that PD-related motor dysfunction selectively affects certain directional axes.

**Fig 2:**
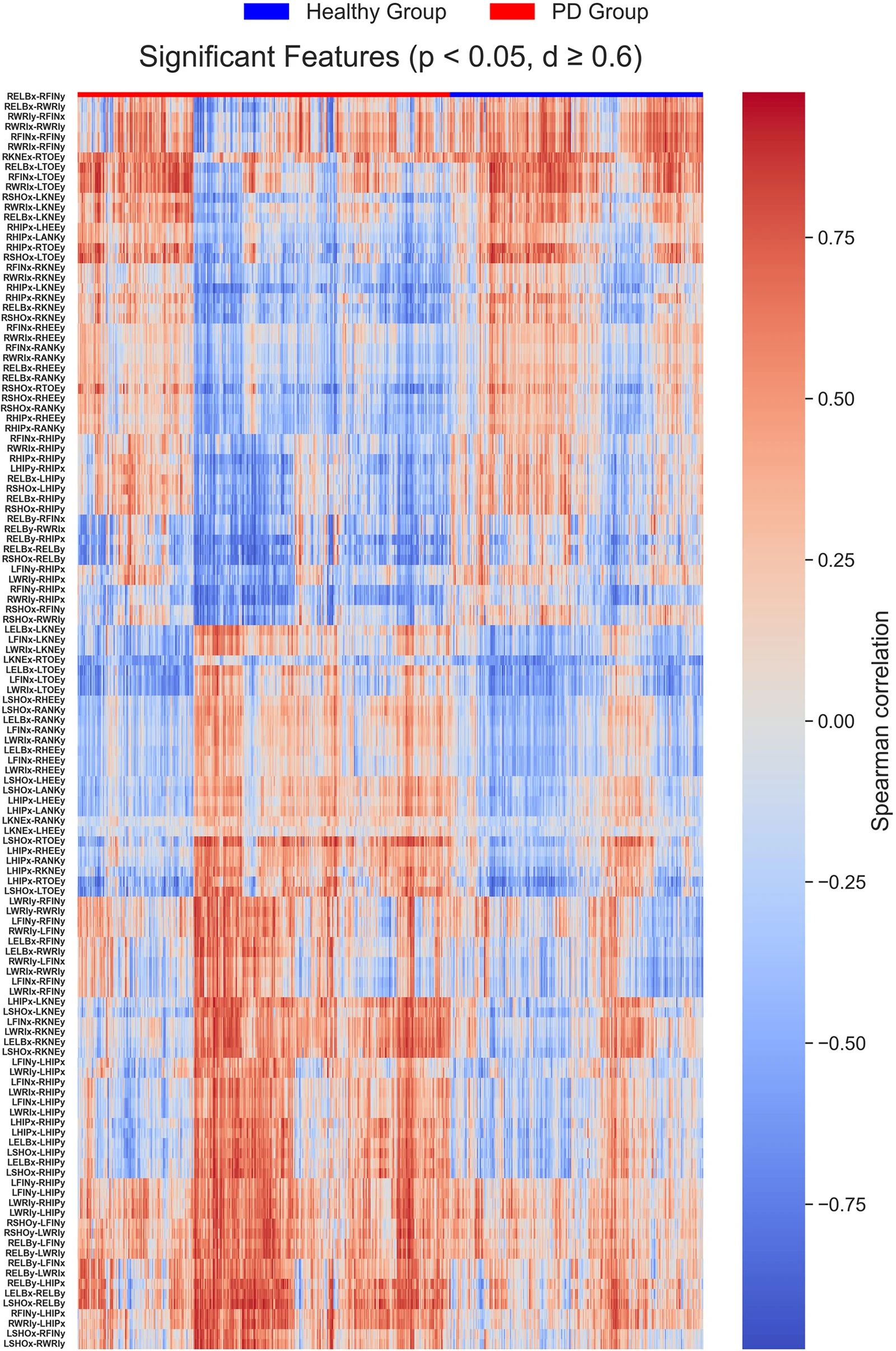
Heatmap of the 125 statistically refined directional body coupling features. Rows represent individual directional coupling pairs (body landmark × axis); columns represent the two groups (PD and HC). Color scale indicates mean absolute Spearman correlation strength, with warmer colors denoting stronger coupling. The color scale bar ranges from 0 (no coupling) to 1 (maximal coupling). The heatmap highlights prominent PD-related strengthening in upper–lower limb interactions and cross-directional components.

In order to validate the coupling patterns on limb segments and their directional changes, we decomposed the 125 identified discriminative coupling factors into three categories: upper–upper limb, lower–lower limb, and cross (upper–lower or lower–upper) limb couplings as summarized in Table 4. Cross-limb couplings constituted the largest proportion, followed by upper–upper and lower–lower connections. The presence of substantial upper–upper and lower–lower within-limb coordination, in addition to the dominant cross-limb interactions, indicates that PD-related changes extend beyond cross-limb couplings to encompass both upper-limb and lower-limb within-segment coordination, reflecting a more global impairment of motor control. S2 Table in S1 File provides detailed information on the 125 discriminative directional body coupling features.

**Table 4:** Distribution of the 125 statistically discriminative directional body coupling features. Values indicate the number of discriminative features. Rows represent the region of the first landmark and columns the region of the second. For cross-limb features, the upper-limb landmark is always listed first.

	Upper limb	Lower limb
Upper limb	32	71
Lower limb	71	22

As we verified that directional information has the potential to unveil complex coupling patterns underlying the time-series gait data, we further decomposed these 125 couplings into four directional categories: horizontal–horizontal (x-x), vertical–vertical (y-y), horizontal–vertical (x-y), and vertical–horizontal (y-x) summarized in Table 5. Importantly, horizontal–vertical and vertical–horizontal couplings are different because of their directional nature. This finding reveals that multi-dimensional movement can be impacted by PD-related defects in inter-limb coordination, which cannot be restricted to a single axis. Moreover, these directional refinements highlight pronounced PD-related strengthening in upper–lower limb interactions and in cross-directional components, suggesting disruptions in multi-planar movement synchronization that are characteristic of PD gait. These axis-specific insights enhance statistical interpretation and offer practical clinical relevance, supporting the potential of directional couplings as biomarkers for PD-related motor dysfunction. S3 Table in S1 File provides all items corresponding to the horizontal and vertical directional coupling factors.

**Table 5:** Horizontal and vertical directional coupling counts among the 125 statistically discriminative features. Rows indicate the axis of the first landmark and columns the axis of the second landmark; off-diagonal cells are direction-ordered and therefore distinct.

	Horizontal	Vertical
Horizontal	0	96
Vertical	17	12

### Improved separation and classification performance using discriminative body coupling factors

To evaluate the discriminative potential of the identified body coupling factors, we examined their ability to separate PD and HC participants in a low-dimensional space using t-SNE [39]. In order to obtain a low-dimensional visualization, we derived two-dimensional coordinates based on the distances among 125 discriminative body coupling factors. Additionally, we also visualized a comparison baseline using 125 randomly selected body coupling factors that were drawn from the non-discriminative body coupling factors. The embedding generated with random, non-discriminative body coupling factors resulted in substantial overlap between PD and HC participants, whereas embeddings derived from discriminative features showed improved group separation with tighter clusters as shown in Fig 3. These findings indicate that the discriminative factors capture more meaningful and structured differences between PD and HC groups.

**Fig 3:**
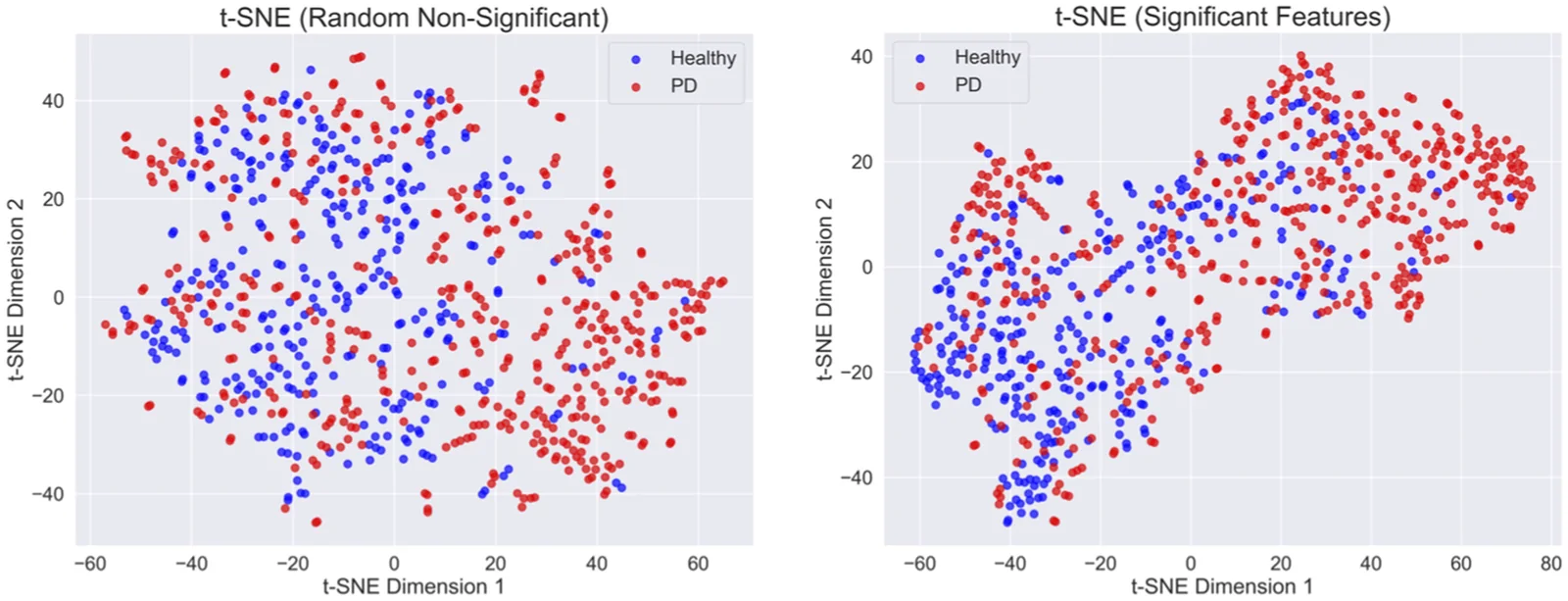
Low-dimensional t-SNE embeddings of PD and HC participants. Horizontal axis: t-SNE Dimension 1; vertical axis: t-SNE Dimension 2. Blue circles: HC participants; red circles: PD participants. The left panel shows randomly selected non-significant body coupling factors, and the right panel shows discriminative body coupling factors, demonstrating improved separation and more coherent clusters.

In order to quantitatively assess the discriminative potential of the identified body coupling factors, we compared clustering quality metrics derived from the low-dimensional coordinates against the true labels of each gait sample. That is, we compared within-cluster sum of squares (WCSS), between-cluster sum of squares (BCSS), and the Dunn Index of the low-dimensional visualization derived from the discriminative body coupling factors against random sampling, as shown in Fig 3. Since WCSS assesses the cohesion of clusters, if the samples within the same label form dense and compact clusters in the low-dimensional visualization, WCSS will result in smaller values. However, WCSS may fail to capture the separation between clusters because it only accounts for the distance of samples from their corresponding cluster centroids. BCSS can effectively quantify the separability of clusters because it computes the distance between each cluster centroid and the global mean of all samples. Note that larger BCSS values generally indicate clear separability. Since WCSS and BCSS can only reflect one side of the coin (i.e., cohesion or separability of clusters), we also compared the Dunn Index of the low-dimensional visualization between discriminative body coupling factors and random sampling because it integrates cohesion and separability in a balanced manner. Additionally, although t-SNE uses the same input data, it yields different visualizations due to its stochastic nature. Hence, we obtained 10 t-SNE visualizations based on different random seeds and averaged the performance metrics to ensure a robust performance comparison.

Based on the averaged performance, we verified that the low-dimensional visualization using discriminative body coupling factors shows slightly higher WCSS, which indicates more scattered clusters compared to the random sampling results. However, this originates from differences in scale in the low-dimensional visualization. That is, as shown in Table 6, since the low-dimensional visualization using discriminative body coupling factors covers a much larger range, direct comparison of WCSS and BCSS can be misleading. Since WCSS indicates cluster compactness and BCSS represents cluster separability, smaller WCSS and larger BCSS can reflect a clearer and more compact low-dimensional visualization across different labels. To this end, we compared the ratio WCSS/BCSS, which yielded 1.15 for random sampling and 1.13 for the discriminative body coupling factors. Consistent results were also obtained from the Dunn Index, which demonstrated that discriminative body coupling improves separability and results in more compact clusters.

**Table 6:** Mean clustering metrics ($\log_{10}$ scale), comparing random non-significant factors and discriminative body coupling factors. Higher BCSS and Dunn Index values indicate improved group separation and cluster compactness.

Metric ($\log_{10}$)	Random (Non-significant)	Discriminative Factors
WCSS	5.986	6.063
BCSS	5.218	5.368
Dunn Index	−0.652	−0.604

To demonstrate the discriminative power of the identified body coupling factors, we compared classification accuracy between the discriminative body coupling factors and randomly selected coupling factors using various machine learning models. It is important to clarify that the primary goal of this study is not to identify the optimal feature sets for machine learning models but report a robust set of body coupling factors both non-directional and directional that hold promise for a wide range of applications, including clinical diagnosis, gait analysis, and other domains involving motor function assessment. However, comparing classification accuracy using machine learning models can demonstrate the effectiveness and potential of the identified body coupling factors.

To this end, based on the random (non-significant) and discriminative body coupling factors, we trained various machine learning models including Support Vector Machine (SVM), Random Forest (RF), Gradient Boosting (GB), Decision Tree (DT), Extreme Gradient Boosting (XGB), and Artificial Neural Network (ANN). We then employed 5-fold cross-validation to obtain a robust and generalizable estimate of classification accuracy.

First, we employed 24 discriminative body coupling factors to train machine learning models, as shown in the left panel of Fig 4 and summarized in Table 7. These six models consistently show clear improvements, with classification accuracy increases exceeding 10%. For instance, SVM showed about a 9% enhancement, while the ANN achieved a 34% improvement in performance. Additionally, with the exception of DT and ANN, the other machine learning models achieved classification accuracies higher than 70%. We also confirmed that discriminative body coupling factors achieved enhanced F1-scores across all machine learning models.

**Table 7:** Precision, recall, and F1-scores (%) for random and discriminative 24-feature sets across models.

Metric (%)	DT		ANN		GB		XGB		RF		SVM	
	Random	Disc.	Random	Disc.	Random	Disc.	Random	Disc.	Random	Disc.	Random	Disc.
Precision	63	73	62	80	62	72	61	72	61	75	68	76
Recall	66	63	42	61	93	82	97	83	94	80	81	79
F1-score	64	67	50	69	74	77	75	77	74	77	74	77

**Fig 4:**
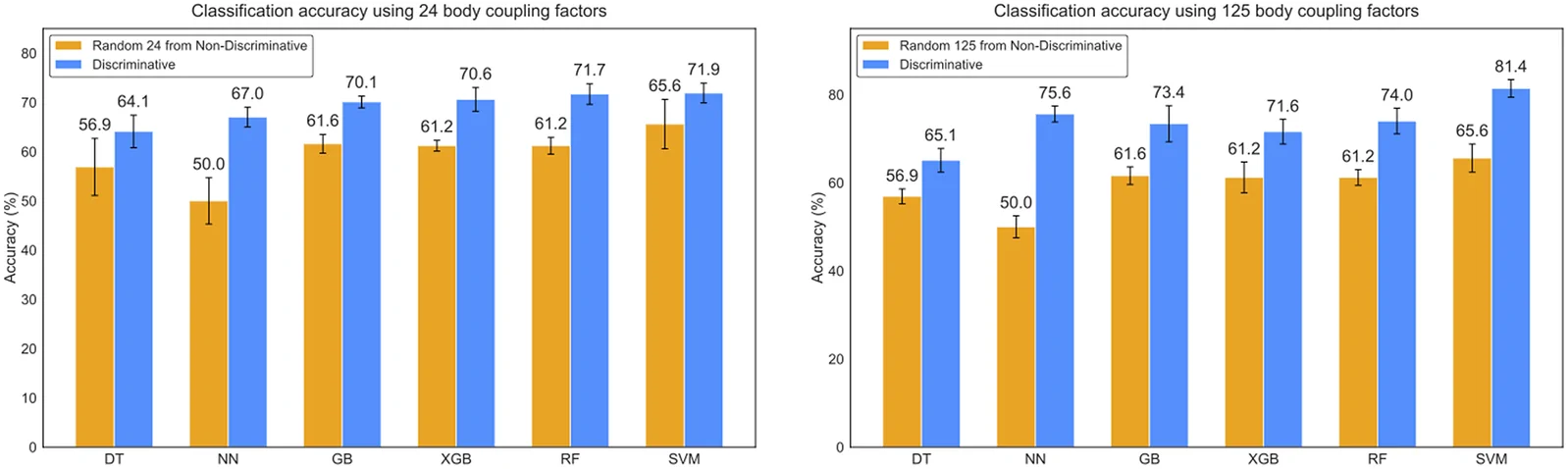
Classification performance using random and discriminative body coupling factors. Bar heights represent mean accuracy (%) across 5-fold cross-validation; error bars indicate ± SD. The left panel shows performance for the 24-feature set (corresponding to Table 7), while the right panel shows performance for the 125-feature set (corresponding to Table 8). Incorporating directional components in the 125-feature set provided consistent performance improvements across all models.

Next, we also trained machine learning models using 125 directional discriminative body coupling factors to evaluate their potential to distinguish between the two groups. Expanding the framework to include 125 directional discriminative body coupling factors further enhanced performance, with precision, recall, and F1-scores increasing by an additional 5–10% compared to the 24-feature configuration, as shown in the right panel of Fig 4 and summarized in Table 8. Moreover, the trained machine learning models achieved higher classification accuracy, which suggests that the 125 directional discriminative body coupling factors possess stronger discriminative power for distinguishing PD from HC groups.

**Table 8:** Precision, recall, and F1-scores (%) for random and discriminative 125-feature sets across models.

Metric (%)	DT		ANN		GB		XGB		RF		SVM	
	Random	Disc.	Random	Disc.	Random	Disc.	Random	Disc.	Random	Disc.	Random	Disc.
Precision	67	80	74	78	62	76	65	76	65	81	72	80
Recall	53	66	57	70	96	81	92	84	91	78	84	82
F1-score	59	72	64	74	75	79	76	80	76	79	78	81

### Association between directional body coupling factors and motor impairment severity in the full PD cohort

To investigate whether the identified discriminative coupling factors reflect not only group-level differences between PD and HC but also clinical motor impairment severity, we computed Spearman rank correlations between the top 10 pre-specified directional body coupling factors and MDS-UPDRS III scores in the full PD cohort (*n* = 121). MDS-UPDRS III was selected as the severity proxy for this analysis because the cohort was predominantly early-stage by both available severity measures: 115 of 121 patients were classified as HY ≤ 2.5, with only six patients classified as HY stage 3 (S1 Fig in S1 File), and disease duration was short and similarly concentrated (mean ± SD: 1.4 ± 1.2 years), leaving neither variable a sufficient range for meaningful correlation or stratification analysis. Accordingly, formal HY stage-stratified analysis was not pursued. The top 10 directional coupling factors were selected based on Cohen’s *d* ranking from the PD versus HC comparison before correlation testing. Per-subject coupling strength values were averaged across repeated walking sequences prior to analysis.

In the full PD cohort, directional body coupling factors showed modest but statistically significant associations with MDS-UPDRS III scores (S2 Fig in S1 File). Complete Spearman coefficients, nominal *p*-values, and FDR-corrected *q*-values for all ten pre-specified features are provided in S4 Table in S1 File. Eight of the ten pre-specified directional coupling factors survived Benjamini–Hochberg FDR correction, and the corresponding scatter plots are presented in S3 Fig in S1 File. Rather than following limb category (upper–upper versus cross-limb), the direction of these associations followed a laterality-based pattern, with cross-body and same-side coupling pairs showing opposite relationships to motor impairment severity.

This predominant pattern, in which stronger cross-body coupling was associated with greater motor impairment, is clinically interpretable. In normal gait, reciprocal cross-body coordination both between the upper and lower limbs and between the two upper limbs is regulated by spinal and supraspinal locomotor networks. In Parkinsonian gait, impaired automatic motor control may reduce independent limb regulation and promote more rigidly coupled whole-body movement patterns. Specifically, the significant cross-body, contralateral coupling pairs were positively associated with MDS-UPDRS III scores, whereas the significant same-side, ipsilateral upper-limb coupling pairs were negatively associated with motor severity (S4 Table in S1 File). This laterality-dependent pattern suggests that increasing motor severity in PD is accompanied by divergent alterations in body coupling: enhanced coupling between opposite sides of the body and reduced same-side upper-limb coupling. This pattern may reflect a shift away from flexible, independently coordinated limb control toward abnormal cross-body synchronization and disrupted within-limb upper-body coordination in Parkinsonian gait. To maintain a rhythmic gait pattern, all body segments must be appropriately coordinated throughout the gait cycle. The excessive cross-body coupling observed in the PD group may disrupt this coordinated rhythmic pattern, thereby potentially increasing the risk of slips, trips, and falls. These findings suggest that patients with PD may have difficulty properly coordinating the movements of individual limbs, potentially due to motor symptoms such as rigidity, one of the cardinal features of PD. These interpretations are based on the observed correlation patterns in the present dataset and should be regarded as hypothesis-generating rather than definitive mechanistic conclusions. The observed positive correlations between cross-body coupling factors and MDS-UPDRS III scores are consistent with the hypothesis that progressive striatal dopamine depletion disrupts automatic inter-limb coordination [40,41]. However, basal ganglia and dopaminergic function were not directly measured in this study; therefore, this interpretation remains inferential and requires future validation using neuroimaging or neurophysiological approaches.

Subgroup analyses were instead limited to exploratory motor subtype-stratified analyses. Although motor subtype is a clinical axis distinct from disease stage, we examined whether the same directional and unified body coupling factors showed subtype-specific associations with MDS-UPDRS III scores, using the Stebbins et al. ratio-based subtype classification criteria [35], to further explore the explanatory potential of these coupling factors across PD motor phenotypes. Among the top 10 unified body coupling features, none reached significance within the PIGD subgroup, where correlations were weak and inconsistent. Within the TD subgroup, however, five unified features showed statistically significant positive associations with MDS-UPDRS III scores after FDR correction: left wrist–right hip, left finger–right hip, left wrist–left hip, left finger–left hip, and right shoulder–left wrist. These associations indicate that, within TD patients specifically, stronger coupling between the wrist or finger and the hip, as well as between the right shoulder and left wrist, was associated with greater motor impairment severity. Full numerical results, including ρ, nominal *p*-values, and FDR-corrected *q*-values for all subtype-stratified analyses, are provided in S5–S8 Tables in S1 File, and the corresponding heatmaps and scatter plots are provided in S4–S8 Figs in S1 File.

We note that this is the only subtype-specific association set surviving FDR correction across both feature sets and both subgroups; because it involves aggregated rather than directional features and arises in the smaller TD subgroup, it should be regarded as an unexpected exploratory result requiring independent replication. Future studies with larger and more balanced subtype cohorts are needed to validate subtype-specific coupling patterns.

It should be emphasized that the present study was designed and statistically powered primarily to identify discriminative body coupling metrics distinguishing PD from HC, rather than to characterize disease-stage- or subtype-related variation in coupling strength. Consistent with this design objective, formal stage-stratified analysis was not pursued in light of the limited power discussed above, and the subtype-stratified findings reported here should be regarded as secondary, hypothesis-generating observations rather than primary study endpoints.

### Limitations

This study demonstrates the feasibility and discriminative value of pose estimation-based body coupling analysis for differentiating PD from HC. Several methodological considerations, outlined below, should nonetheless be taken into account when interpreting these findings.

First, gait data were acquired using 2D pose estimation from a single lateral camera, restricting the analysis to x- and y-axis landmark trajectories. The absence of depth information along the z-axis limited the ability to characterize three-dimensional gait features, including anterior-posterior movement, transverse-plane trunk rotation, and pelvic axial rotation. These 3D movement components may contain additional clinically relevant information and could represent more sensitive biomarkers of PD-related gait impairment. Future studies should incorporate stereo cameras or multi-view 3D pose estimation to provide a more comprehensive characterization of three-dimensional body coupling patterns in PD gait.

Second, the walking protocol involved only three to four steps per trial from a standing start. This short protocol was deliberately adopted to accommodate clinical constraints, including fall risk, fatigue susceptibility, and limited corridor space in a hospital setting. Because participants began walking from quiet standing, gait-initiation phases were naturally included and were not explicitly separated from subsequent short-distance walking. This design may limit the characterization of pure steady-state gait coupling and may introduce some kinematic variability related to movement initiation. However, gait initiation is itself clinically relevant in PD because difficulty initiating movement is a recognized feature of parkinsonian motor impairment [42,43]. In addition, repeated trials yielded 486 single-direction walking sequences in the PD group and 330 in the HC group, allowing coupling estimates to be averaged across multiple sequences. Future studies should incorporate longer walkways and explicit segmentation of gait-initiation and steady-state phases to independently characterize these components.

Third, several aspects of cohort composition and representativeness should be noted. (a) Sex distribution was not matched between groups: the PD group included 53 female and 68 male participants, whereas the HC group included 50 female and 32 male participants. Given that the PD cohort was predominantly mild-to-moderate, with 95% of patients classified as HY ≤2.5, sex-related differences in spatiotemporal gait parameters at this severity range are reported to be minimal [44]. Nonetheless, sex-matched cohort designs are recommended for future studies. (b) Medication status, including dopaminergic ON and OFF state, was not recorded. Because dopaminergic therapy can influence gait performance, future studies should evaluate body-coupling features under defined medication states, ideally through within-subject ON/OFF comparisons, to clarify the extent to which dopaminergic state modulates these coupling patterns. (c) The cohort contains few patients with advanced PD (HY 3, *n* = 6), and recruitment was restricted to early-stage disease (duration ≤3 years), limiting generalizability to later stages and patients with longer disease histories. Additionally, because the narrow disease-duration range (≤3 years, per inclusion criteria) limited variance within the cohort, examining disease duration itself as a continuous correlate of coupling strength was not feasible in the present study. (d) Subtype-stratified analyses were exploratory and limited by smaller subgroup sample sizes compared with the full PD cohort. Therefore, subtype-specific findings should be interpreted cautiously and require validation in larger and more balanced subtype cohorts before definitive subtype-specific conclusions can be drawn. We emphasize that this study was primarily designed and powered to establish discriminative body coupling metrics differentiating PD from HC, rather than to characterize disease-stage- or subtype-specific differences in coupling scores. Accordingly, formal stage-stratified analysis was not attempted given the limited power noted above, and the subtype-stratified analyses presented here are offered as secondary, hypothesis-generating extensions rather than primary study endpoints.

Fourth, this study is cross-sectional and cannot address disease progression. Longitudinal validation is required to confirm the utility of these coupling metrics for monitoring disease trajectories over time.

Finally, basal ganglia and dopaminergic function were not directly measured in this study. Therefore, the pathophysiological interpretations linking coupling abnormalities to striatal dysfunction remain inferential, and direct neurophysiological validation using neuroimaging or electrophysiology is required in future work.

## Conclusion

This study presents a comprehensive framework for quantifying and analyzing whole-body coupling dynamics to differentiate individuals with PD from HC. Based on the human pose estimation framework, we extracted time-series coordinate changes of key body points during walking and quantitatively estimated coupling strength among key body points using body coupling scores derived from correlation coefficients. Through statistical analysis, we identified discriminative body coupling factors that meet statistical significance and ensure meaningful differences between the two groups. We further extended these discriminative factors by incorporating directional information, providing more granular insights into whole-body coupling differences between PD and HC groups.

The comprehensive analysis highlights that the identified discriminative body coupling factors exhibit clear differences in coupling strength between PD and HC groups. Visualization through heatmaps revealed distinctive coupling patterns in the PD group, particularly in cross-limb and cross-directional interactions, emphasizing the altered motor coordination patterns associated with the disease. Complementary t-SNE projections demonstrated clear group-level separations, supporting the discriminative potential of the identified coupling factors. These findings were further reinforced by machine learning evaluations, where models trained on the discriminative coupling features consistently achieved higher precision, recall, and F1-scores compared to those trained on non-significant random features.

Correlation analyses between the top 10 pre-specified directional body coupling factors and MDS-UPDRS III scores demonstrated modest but statistically significant associations in the full PD cohort (*n* = 121), with 8 of 10 directional factors surviving FDR correction (ρ=0.27–0.31). These findings provide initial evidence that directional body coupling metrics carry clinically relevant information beyond binary PD versus HC discrimination and may serve as potential digital biomarkers for quantifying PD-related motor impairment severity. The predominant pattern, in which stronger cross-limb and cross-directional coupling was associated with greater motor impairment, is consistent with disrupted automatic inter-limb coordination in PD; however, direct neurophysiological validation is required because basal ganglia and dopaminergic function were not measured in this study. Subtype-stratified analyses were conducted only as exploratory supplementary analyses. Although nominal subtype-specific trends were observed, larger and more balanced subtype cohorts are required before definitive subtype-specific conclusions can be drawn.

Overall, our integrated framework combining statistical filtering, interpretable visualization, and predictive modeling offers a scalable, non-invasive, and cost-effective tool for PD gait assessment. Future work should extend this approach to longitudinal datasets to track disease progression, incorporate 3D pose estimation to recover transverse-plane dynamics, validate findings in sex-matched and medication-controlled cohorts, validate exploratory subtype-specific trends in larger and more balanced subtype cohorts, obtain direct neurophysiological validation of the basal ganglia interpretation, and confirm clinical utility in real-world diagnostic and monitoring applications.

## Supporting information

S1 File Supplementary figures and tables. This file contains Figures S1–S8 and Tables S1–S8, providing additional results on differential and directional body coupling analyses, discriminative coupling features, and exploratory associations with MDS-UPDRS III scores.
(PDF)
