# Supplementary material for: Differential and directional body coupling analysis in Parkinsonian gait
Source: PLoS One. 2026 Nov 4;21(11):e0361959. doi: 10.1371/journal.pone.0361959 (PMC13734472; doi:10.1371/journal.pone.0361959)
Supplement: Supplementary file 1 [file pone.0361959.s001.pdf]

# Supplementary Material for Differential and Directional Body Coupling Analysis in Parkinsonian Gait

Arifa Zahir[1], Jaehong Yu[2], Jin-Sun Jun[3], Kiwon Park[1]*, Ryul Kim[4]*, and Hyundoo Jeong[1]*
[1] Department of Biomedical and Robotics Engineering, Incheon National University, 119 Academy-ro, Yeonsu-gu, Songdo-dong, Incheon 22012, Republic of Korea
[2] Department of Industrial and Management Engineering, Incheon National University, 119 Academy-ro, Yeonsu-gu, Songdo-dong, Incheon 22012, Republic of Korea
[3] Department of Neurology, Kangnam Sacred Heart Hospital, Hallym University College of Medicine, Seoul, Republic of Korea
[4] Department of Neurology, Seoul Metropolitan Government–Seoul National University Boramae Medical Center, Seoul National University College of Medicine, Seoul, Republic of Korea

Correspondence: hdj@inu.ac.kr

## Contents

**Supplementary Methods** **3**

Pre-specification of coupling factors for full PD cohort severity correlation analysis . . . . 3

Exploratory motor subtype classification and subtype-stratified analysis . . . . . . . . . . 3

Summary of verified supplementary correlation results . . . . . . . . . . . . . . . . . . . . 4

## List of Tables

S1 The 24 discriminative unified body coupling features. . . . . . . . . . . . . . . . . . . . 5

S2 The 125 discriminative directional body coupling features . . . . . . . . . . . . . . . . 6

S3 Horizontal and vertical directional coupling breakdown. . . . . . . . . . . . . . . . . . 7

S4 Full PD cohort correlations between top 10 directional features and MDS-UPDRS III 8

S5 Exploratory PIGD subgroup correlations: top 10 directional features with MDS-UPDRS III . . . . . . . . . . . . . . . . . . . . . . . . . . . . . . . . . . . . . . . . 10

S6 Exploratory TD subgroup correlations: top 10 directional features with MDS-UPDRS III . . . . . . . . . . . . . . . . . . . . . . . . . . . . . . . . . . . . . . . . 11

S7 Exploratory PIGD subgroup correlations: top 10 unified features with MDS-UPDRS III . . . . . . . . . . . . . . . . . . . . . . . . . . . . . . . . . . . . . . . . 12

S8 Exploratory TD subgroup correlations: top 10 unified features with MDS-UPDRS III 13

# List of Figures


S1 Distribution of Hoehn and Yahr stages in the PD cohort. . . . . . . . . . . . . . . . . . 14
S2 Full PD cohort heatmap: top 10 directional features with MDS-UPDRS III . . . . . 15
S3 Continued . . . . . . . . . . . . . . . . . . . . . . . . . . . . . . . . . . . . . . . . . . 16
S3 Full PD cohort scatter plots: top 10 directional features with MDS-UPDRS III . . . 17
S4 Exploratory subtype-stratified heatmap: top 10 directional and unified features . . . 18
S5 Continued . . . . . . . . . . . . . . . . . . . . . . . . . . . . . . . . . . . . . . . . . . 19
S5 Exploratory PIGD subgroup scatter plots: top 10 directional features . . . . . . . . . 20
S6 Continued . . . . . . . . . . . . . . . . . . . . . . . . . . . . . . . . . . . . . . . . . . 21
S6 Exploratory TD subgroup scatter plots: top 10 directional features . . . . . . . . . . 22
S7 Continued . . . . . . . . . . . . . . . . . . . . . . . . . . . . . . . . . . . . . . . . . . 23
S7 Exploratory PIGD subgroup scatter plots: top 10 unified features . . . . . . . . . . . 24
S8 Continued . . . . . . . . . . . . . . . . . . . . . . . . . . . . . . . . . . . . . . . . . . 25
S8 Exploratory TD subgroup scatter plots: top 10 unified features . . . . . . . . . . . . 26

## Supplementary Methods

### Pre-specification of coupling factors for full PD cohort severity correlation analysis

To evaluate whether gait-coupling abnormalities were associated with clinical motor impairment severity, a full PD cohort correlation analysis was performed using the verified PD cohort ($n = 121$). To avoid outcome-driven feature selection, coupling factors examined in the correlation analysis were pre-specified before association testing. From the set of 125 discriminative directional body coupling features, the 10 factors with the largest PD versus HC effect sizes, quantified by Cohen's $d$, were selected. This selection criterion was based on diagnostic group separation and was independent of MDS-UPDRS III severity scores. Spearman rank correlation analyses were then performed between these pre-specified directional body coupling features and MDS-UPDRS III scores in the full PD cohort, followed by Benjamini–Hochberg FDR correction across the 10 tested features.

### Exploratory motor subtype classification and subtype-stratified analysis

Motor subtype analyses were conducted as exploratory supplementary analyses to examine whether coupling–severity relationships differed between clinical motor subtypes.

Motor subtypes were classified according to the MDS-UPDRS ratio-based criteria described by Stebbins et al. [1]. The tremor score was calculated as the arithmetic mean of tremor-related MDS-UPDRS III items, and the PIGD score was calculated as the arithmetic mean of postural-instability and gait-related MDS-UPDRS III items. The TD/PIGD ratio was then computed for each patient. Patients were classified as tremor-dominant (TD) when the TD/PIGD ratio was $\geq 1.15$, as postural instability and gait difficulty (PIGD) when the TD/PIGD ratio was $\leq 0.90$, and as Indeterminate when $0.90 <$ TD/PIGD ratio $< 1.15$. Among the 121 PD patients, 49 were classified as TD, 53 as PIGD, and 19 as Indeterminate. The full PD cohort analysis included all 121 patients, whereas subtype-stratified analyses were restricted to the TD and PIGD subgroups.

For subtype-stratified analyses, Spearman rank correlations were calculated between the pre-specified coupling features and MDS-UPDRS III scores within the PIGD and TD subgroups. Benjamini–Hochberg FDR correction was applied within each tested subtype-specific feature set. Because subgroup sizes were limited and imbalanced, these results should be interpreted as exploratory trends requiring confirmation in larger and more balanced subtype cohorts.

## Summary of verified supplementary correlation results

In the full PD cohort, 8 of the 10 pre-specified directional body coupling features were significantly associated with MDS-UPDRS III scores after FDR correction, with modest effect sizes among the significant features $|\rho| \approx 0.27$–$0.31$. These full-cohort results support the potential clinical relevance of directional coupling metrics as digital measures related to motor impairment severity. Subtype-stratified analyses are presented as exploratory supplementary analyses. These findings should be interpreted with consideration of limited and imbalanced subgroup sample sizes, and larger, independent, and more balanced cohorts are needed to confirm subtype-specific coupling patterns before subtype-specific conclusions can be drawn.

## Supplementary Tables

Table S1: **The 24 discriminative unified body coupling features.** Full listing of the 24 discriminative unified body coupling features grouped by anatomical limb-pair category. The retained unified coupling factors were mainly distributed across upper–upper and upper–lower/lower–upper interactions, while no lower–lower unified coupling factor was retained. Because unified coupling is non-directional, upper–lower and lower–upper interactions are summarized in a single combined category to avoid duplicate listing.

| **Group** | **Count** | **Features** |
|---|---|---|
| Upper–Upper | 8 | RELB–LFIN, RELB–LWRI, RSHO–LFIN, RSHO–LWRI, LSHO–RFIN, LSHO–RWRI, LELB–RELB, LSHO–RELB |
| Upper–Lower / Lower–Upper | 16 | RFIN–RHIP, RWRI–RHIP, LFIN–LHIP, LWRI–LHIP, LWRI–RHIP, LFIN–RHIP, RFIN–LHIP, RWRI–LHIP, LSHO–RHIP, LSHO–LHIP, LELB–RHIP, LELB–LHIP, RSHO–RHIP, RSHO–LHIP, RELB–RHIP, RELB–LHIP |
| Lower–Lower | 0 | — |

Table S2: **The 125 discriminative directional body coupling features.** Full listing of the 125 discriminative directional body coupling features grouped by anatomical limb-pair category. The retained directional coupling factors were distributed across upper–upper, upper–lower/lower–upper, and lower–lower interactions. Because cross-limb interactions describe coupling between upper- and lower-body regions, upper–lower and lower–upper interactions are summarized in a single combined category to avoid duplicate listing. Counts sum to 125.

| **Group** | **Count** | **Features** |
|---|---|---|
| Upper–Upper | 32 | RFINx–RFINy, RWRIx–RFINy, RWRIx–RWRIy, RELBx–RFINy, RELBx–RWRIy, LFINx–RFINy, LWRIx–RFINy, LWRIx–RWRIy, LELBx–RFINy, LELBx–RWRIy, LELBx–RELBy, LSHOx–RELBy, LSHOx–RFINy, LSHOx–RWRIy, RSHOx–RFINy, RSHOx–RWRIy, RELBx–RELBy, RSHOx–RELBy, RWRIy–RFINx, RWRIy–LFINx, RELBy–LFINx, RELBy–LWRIx, RELBy–RFINx, RELBy–RWRIx, RELBy–LFINy, RELBy–LWRIy, RSHOy–LFINy, RSHOy–LWRIy, LFINy–RFINy, RWRIy–LFINy, LWRIy–RFINy, LWRIy–RWRIy |
| Upper–Lower / Lower–Upper | 71 | RFINx–LTOEy, RWRIx–LTOEy, RELBx–LTOEy, RWRIx–LKNEy, RELBx–LKNEy, LSHOx–RTOEy, LSHOx–LTOEy, LSHOx–LKNEy, LSHOx–LHEEy, LSHOx–LANKy, LELBx–RKNEy, LSHOx–RKNEy, LFINx–RKNEy, LWRIx–RKNEy, LFINx–LTOEy, LWRIx–LTOEy, LELBx–LTOEy, LFINx–LKNEy, LWRIx–LKNEy, LELBx–LKNEy, LFINx–RHEEy, LWRIx–RHEEy, LELBx–RHEEy, LFINx–RANKy, LWRIx–RANKy, LELBx–RANKy, LSHOx–RHEEy, LSHOx–RANKy, LELBx–RHIPy, LSHOx–RHIPy, LELBx–LHIPy, LSHOx–LHIPy, LFINx–LHIPy, LWRIx–LHIPy, LFINx–RHIPy, LWRIx–RHIPy, RSHOx–RTOEy, RSHOx–RHEEy, RSHOx–RANKy, RSHOx–LTOEy, RELBx–RKNEy, RSHOx–RKNEy, RSHOx–LKNEy, RFINx–RKNEy, RWRIx–RKNEy, RELBx–RHEEy, RELBx–RANKy, RFINx–RANKy, RWRIx–RANKy, RFINx–RHEEy, RWRIx–RHEEy, RELBx–RHIPy, RSHOx–RHIPy, RELBx–LHIPy, RSHOx–LHIPy, RFINx–RHIPy, RWRIx–RHIPy, RELBy–LHIPx, LFINy–LHIPx, LWRIy–LHIPx, RFINy–LHIPx, RWRIy–LHIPx, RFINy–RHIPx, RWRIy–RHIPx, LFINy–RHIPx, LWRIy–RHIPx, RELBy–RHIPx, LWRIy–RHIPy, LWRIy–LHIPy, LFINy–RHIPy, LFINy–LHIPy |
| Lower–Lower | 22 | RKNEx–RTOEy, LKNEx–RANKy, LKNEx–LHEEy, LHIPx–RTOEy, LHIPx–RKNEy, LHIPx–RHEEy, LHIPx–RANKy, LHIPx–LKNEy, LHIPx–LHEEy, LHIPx–LANKy, LHIPx–RHIPy, LHIPx–LHIPy, RHIPx–RHEEy, RHIPx–RANKy, RHIPx–RTOEy, RHIPx–RKNEy, RHIPx–LKNEy, RHIPx–LHEEy, RHIPx–LANKy, RHIPx–RHIPy, LKNEx–RTOEy, LHIPy–RHIPx |

Table S3: **Horizontal and vertical directional coupling breakdown.** Horizontal/vertical directional composition of the 125 discriminative directional body coupling features. Most retained features involved horizontal–vertical coupling, whereas no horizontal–horizontal feature was retained. This pattern suggests that cross-directional coordination contributed substantially to PD–HC discrimination.

| Group | Count | Features |
|---|---|---|
| Horizontal–Horizontal | 0 | — |
| Horizontal–Vertical | 96 | RFINx–LTOEy, RWRIx–LTOEy, RELBx–LTOEy, RWRIx–LKNEy, RELBx–LKNEy, RFINx–RFINy, RWRIx–RFINy, RWRIx–RWRIy, RELBx–RFINy, RELBx–RWRIy, RKNEx–RTOEy, LKNEx–RANKy, LKNEx–LHEEy, LFINx–RFINy, LWRIx–RFINy, LWRIx–RWRIy, LELBx–RFINy, LELBx–RWRIy, LHIPx–RTOEy, LHIPx–RKNEy, LHIPx–RHEEy, LHIPx–RANKy, LSHOx–RTOEy, LSHOx–LTOEy, LHIPx–LKNEy, LSHOx–LKNEy, LHIPx–LHEEy, LHIPx–LANKy, LSHOx–LHEEy, LSHOx–LANKy, LELBx–RKNEy, LSHOx–RKNEy, LFINx–RKNEy, LWRIx–RKNEy, LFINx–LTOEy, LWRIx–LTOEy, LELBx–LTOEy, LFINx–LKNEy, LWRIx–LKNEy, LELBx–LKNEy, LFINx–RHEEy, LWRIx–RHEEy, LELBx–RHEEy, LFINx–RANKy, LWRIx–RANKy, LELBx–RANKy, LSHOx–RHEEy, LSHOx–RANKy, LELBx–RELBy, LSHOx–RELBy, LSHOx–RFINy, LSHOx–RWRIy, LELBx–RHIPy, LSHOx–RHIPy, LELBx–LHIPy, LSHOx–LHIPy, LHIPx–RHIPy, LHIPx–LHIPy, LFINx–LHIPy, LWRIx–LHIPy, LFINx–RHIPy, LWRIx–RHIPy, RSHOx–RTOEy, RHIPx–RHEEy, RHIPx–RANKy, RSHOx–RHEEy, RSHOx–RANKy, RHIPx–RTOEy, RSHOx–LTOEy, RELBx–RKNEy, RSHOx–RKNEy, RHIPx–RKNEy, RSHOx–LKNEy, RHIPx–LKNEy, RFINx–RKNEy, RWRIx–RKNEy, RELBx–RHEEy, RELBx–RANKy, RFINx–RANKy, RWRIx–RANKy, RFINx–RHEEy, RWRIx–RHEEy, RHIPx–LHEEy, RHIPx–LANKy, RSHOx–RFINy, RSHOx–RWRIy, RELBx–RELBy, RSHOx–RELBy, RELBx–RHIPy, RSHOx–RHIPy, RELBx–LHIPy, RSHOx–LHIPy, RHIPx–RHIPy, RFINx–RHIPy, RWRIx–RHIPy, LKNEx–RTOEy |
| Vertical–Horizontal | 17 | RWRIy–RFINx, RWRIy–LFINx, RELBy–LHIPx, RELBy–LFINx, RELBy–LWRIx, LFINy–LHIPx, LWRIy–LHIPx, RFINy–LHIPx, RWRIy–LHIPx, RFINy–RHIPx, RWRIy–RHIPx, LFINy–RHIPx, LWRIy–RHIPx, RELBy–RHIPx, RELBy–RFINx, RELBy–RWRIx, LHIPy–RHIPx |
| Vertical–Vertical | 12 | RELBy–LFINy, RELBy–LWRIy, RSHOy–LFINy, RSHOy–LWRIy, LWRIy–RHIPy, LWRIy–LHIPy, LFINy–RHIPy, LFINy–LHIPy, LFINy–RFINy, RWRIy–LFINy, LWRIy–RFINy, LWRIy–RWRIy |

Table S4: **Full PD cohort correlations between the top 10 pre-specified directional body coupling features and MDS-UPDRS III scores.** Spearman rank correlations between the top 10 pre-specified directional body coupling features, drawn from the 125 discriminative directional body coupling features, and MDS-UPDRS III scores in the full PD cohort ($n = 121$). Eight of the ten features remained statistically significant after Benjamini–Hochberg FDR correction. $\rho$ denotes Spearman rank correlation coefficient; $p$ denotes nominal significance; and $q$ denotes FDR-corrected significance. Bold values indicate statistically significant associations after FDR correction ($q < 0.05$).

| **Coupling factor** | **Spearman** $\rho$ | $p$ | **FDR** $q$ |
|---|---|---|---|
| **LELBx–RHEEy** | **0.281** | **0.0018** | **0.0026** |
| LELBx–RHIPy | 0.141 | 0.1236 | 0.1236 |
| **LELBx–RWRIy** | **0.296** | **0.0010** | **0.0025** |
| **LFINx–RHEEy** | **0.307** | **0.0006** | **0.0025** |
| **LSHOx–RFINy** | **0.295** | **0.0010** | **0.0025** |
| LSHOx–RHIPy | 0.148 | 0.1052 | 0.1169 |
| **LSHOx–RWRIy** | **0.274** | **0.0024** | **0.0029** |
| **LWRIx–RHEEy** | **0.302** | **0.0008** | **0.0025** |
| **RSHOx–RFINy** | **−0.286** | **0.0015** | **0.0026** |
| **RSHOx–RWRIy** | **−0.282** | **0.0017** | **0.0026** |

**Anatomical interpretation of Table S4.** The correlation results showed a laterality-dependent pattern: cross-body, contralateral coupling pairs tended to show positive correlations with MDS-UPDRS III scores, whereas same-side, ipsilateral upper-limb coupling pairs showed negative correlations. Among the ten pre-specified directional coupling factors, six significant cross-body pairs, including three cross-limb pairs (left elbow–right heel, left finger–right heel, and left wrist–right heel) and three upper–upper pairs (left elbow–right wrist, left shoulder–right finger, and left shoulder–right wrist), were positively associated with MDS-UPDRS III scores. In contrast, the two significant same-side upper-limb pairs (right shoulder–right finger and right shoulder–right wrist) were negatively associated with MDS-UPDRS III scores. The remaining two cross-body pairs (left elbow–right hip and left shoulder–right hip) showed positive correlations but did not survive FDR correction. Notably, these two non-significant cross-body pairs involved hip-related couplings, whereas the significant cross-body associations predominantly involved distal-limb landmarks such as the heel, wrist, or finger. Overall, these findings suggest laterality-dependent alterations in body coupling, with enhanced coupling between opposite sides of the body and reduced same-side upper-limb coupling as motor severity increases in PD. This pattern may reflect reduced independent limb control, increasingly rigid cross-body coordination, and disrupted within-limb upper-body coordination. Because this interpretation is based on a limited number of representative coupling factors, it should be regarded as hypothesis-generating.

Table S5: **Exploratory PIGD subgroup correlations between the top 10 pre-specified directional body coupling features and MDS-UPDRS III scores.** Exploratory Spearman rank correlations between the top 10 pre-specified directional body coupling features and MDS-UPDRS III scores within the PIGD subgroup ($n = 53$). None of these features survived Benjamini–Hochberg FDR correction. $\rho$ denotes Spearman rank correlation coefficient; $p$ denotes nominal significance; and $q$ denotes FDR-corrected significance. These subtype-stratified findings are exploratory and require confirmation in larger, balanced subtype cohorts.

| **Coupling factor** | **Spearman** $\rho$ | $p$ | **FDR** $q$ |
|---|---|---|---|
| RSHOx–RWRIy | −0.232 | 0.0940 | 0.2351 |
| RSHOx–RFINy | −0.198 | 0.1546 | 0.2577 |
| LELBx–RHEEy | 0.246 | 0.0752 | 0.2351 |
| LSHOx–RWRIy | 0.179 | 0.1985 | 0.2735 |
| LWRIx–RHEEy | 0.251 | 0.0698 | 0.2351 |
| LELBx–RWRIy | 0.206 | 0.1397 | 0.2577 |
| LFINx–RHEEy | 0.248 | 0.0732 | 0.2351 |
| LSHOx–RHIPy | 0.030 | 0.8316 | 0.8316 |
| LELBx–RHIPy | −0.036 | 0.7959 | 0.8316 |
| LSHOx–RFINy | 0.172 | 0.2188 | 0.2735 |

Table S6: **Exploratory TD subgroup correlations between the top 10 pre-specified directional body coupling features and MDS-UPDRS III scores.** Exploratory Spearman rank correlations between the top 10 pre-specified directional body coupling features and MDS-UPDRS III scores within the TD subgroup ($n = 49$). Although some features showed nominal associations, none survived Benjamini–Hochberg FDR correction at $q < 0.05$. $\rho$ denotes Spearman rank correlation coefficient; $p$ denotes nominal significance; and $q$ denotes FDR-corrected significance. These results require validation in a larger TD cohort.

| **Coupling factor** | **Spearman** $\rho$ | $p$ | **FDR** $q$ |
|---|---|---|---|
| RSHOx–RWRIy | −0.228 | 0.1156 | 0.1651 |
| RSHOx–RFINy | −0.261 | 0.0706 | 0.1402 |
| LELBx–RHEEy | 0.204 | 0.1593 | 0.1991 |
| LSHOx–RWRIy | 0.295 | 0.0395 | 0.1315 |
| LWRIx–RHEEy | 0.249 | 0.0841 | 0.1402 |
| LELBx–RWRIy | 0.361 | 0.0109 | 0.0561 |
| LFINx–RHEEy | 0.262 | 0.0693 | 0.1402 |
| LSHOx–RHIPy | 0.142 | 0.3303 | 0.3303 |
| LELBx–RHIPy | 0.159 | 0.2754 | 0.3060 |
| LSHOx–RFINy | 0.359 | 0.0112 | 0.0561 |

Table S7: **Exploratory PIGD subgroup correlations between the top 10 pre-specified unified body coupling features and MDS-UPDRS III scores.** Exploratory Spearman rank correlations between the top 10 pre-specified unified body coupling features and MDS-UPDRS III scores within the PIGD subgroup ($n = 53$). Correlations were weak and inconsistent, and none survived Benjamini–Hochberg FDR correction. $\rho$ denotes Spearman rank correlation coefficient; $p$ denotes nominal significance; and $q$ denotes FDR-corrected significance. These findings should be interpreted cautiously.

| **Coupling factor** | **Spearman** $\rho$ | $p$ | **FDR** $q$ |
|---|---|---|---|
| RSHO–RHIP | −0.044 | 0.7559 | 0.8633 |
| LWRI–RHIP | −0.130 | 0.3552 | 0.8633 |
| LFIN–RHIP | −0.013 | 0.9287 | 0.9287 |
| LWRI–LHIP | −0.145 | 0.3019 | 0.8633 |
| RELB–LWRI | −0.103 | 0.4633 | 0.8633 |
| RSHO–LFIN | −0.051 | 0.7155 | 0.8633 |
| LFIN–LHIP | 0.040 | 0.7770 | 0.8633 |
| LSHO–RELB | 0.141 | 0.3141 | 0.8633 |
| LELB–RHIP | −0.150 | 0.2843 | 0.8633 |
| RSHO–LWRI | −0.051 | 0.7180 | 0.8633 |

Table S8: **Exploratory TD subgroup correlations between the top 10 pre-specified unified body coupling features and MDS-UPDRS III scores.** Exploratory Spearman rank correlations between the top 10 pre-specified unified body coupling features and MDS-UPDRS III scores within the TD subgroup ($n = 49$). Several features showed positive associations after Benjamini–Hochberg FDR correction; however, these subtype-stratified findings should be interpreted cautiously because of the limited TD subgroup sample size. $\rho$ denotes Spearman rank correlation coefficient; $p$ denotes nominal significance; and $q$ denotes FDR-corrected significance. Bold values indicate associations with $q < 0.05$, which require validation in larger, independent, and more balanced subtype cohorts before subtype-specific conclusions can be drawn.

| **Coupling factor** | **Spearman $\rho$** | $p$ | **FDR $q$** |
|---|---|---|---|
| RSHO–RHIP | −0.036 | 0.8076 | 0.8076 |
| **LWRI–RHIP** | **0.338** | **0.0174** | **0.0441** |
| **LFIN–RHIP** | **0.326** | **0.0220** | **0.0441** |
| **LWRI–LHIP** | **0.355** | **0.0122** | **0.0441** |
| RELB–LWRI | 0.220 | 0.1281 | 0.1831 |
| RSHO–LFIN | 0.301 | 0.0358 | 0.0597 |
| **LFIN–LHIP** | **0.335** | **0.0186** | **0.0441** |
| LSHO–RELB | −0.043 | 0.7676 | 0.8076 |
| LELB–RHIP | 0.187 | 0.1983 | 0.2479 |
| **RSHO–LWRI** | **0.338** | **0.0175** | **0.0441** |

# Supplementary Figures

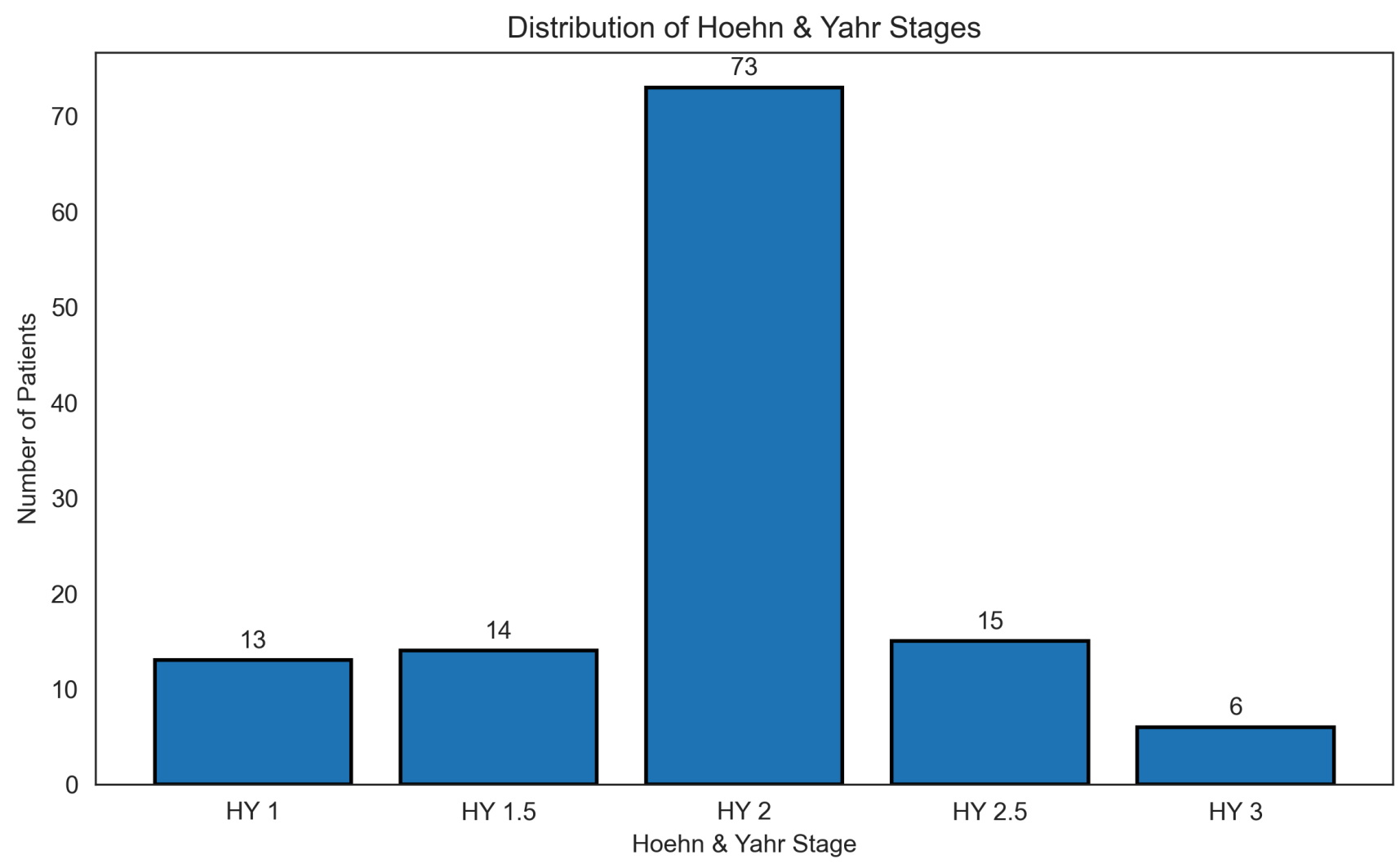


Figure S1: **Distribution of Hoehn and Yahr stages in the PD cohort ($n = 121$).** Bar chart showing the number of PD patients at each HY stage. The distribution was HY stage 1, $n = 13$; HY stage 1.5, $n = 14$; HY stage 2, $n = 73$; HY stage 2.5, $n = 15$; and HY stage 3, $n = 6$. Most patients were classified as mild-to-moderate PD, with 115 of 121 patients having HY stage $\leq 2.5$.

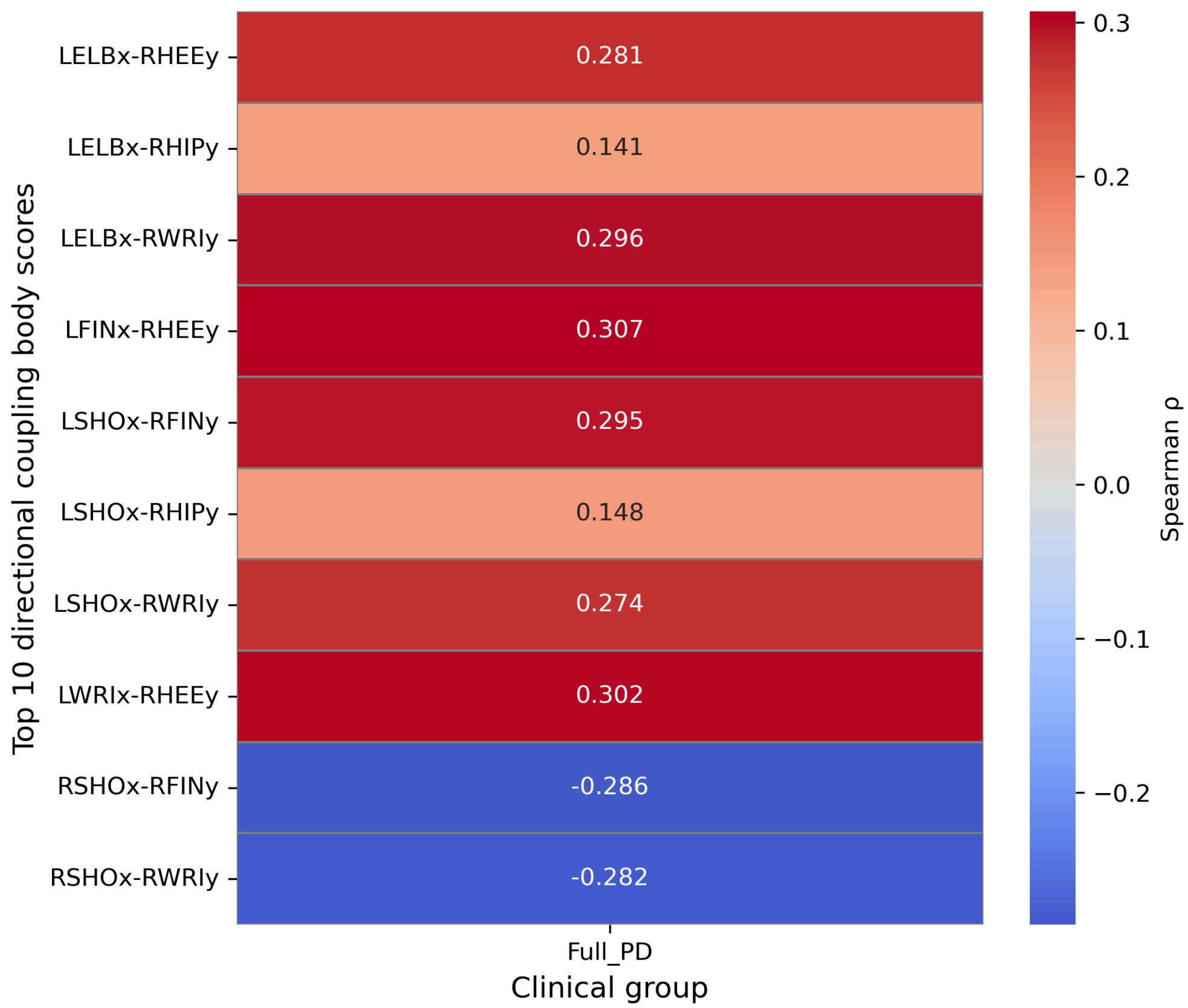


Figure S2: **Full PD cohort heatmap of Spearman correlations between the top 10 directional coupling features and MDS-UPDRS III scores ($n = 121$).** Rows represent the top 10 pre-specified features drawn from the 125 discriminative directional body coupling features. The single column represents the full PD cohort. Color indicates Spearman $\rho$, with warmer colors representing positive correlations and cooler colors representing negative correlations. Eight of the ten features survived FDR correction, supporting the potential clinical relevance of directional coupling metrics as digital measures of motor impairment severity.

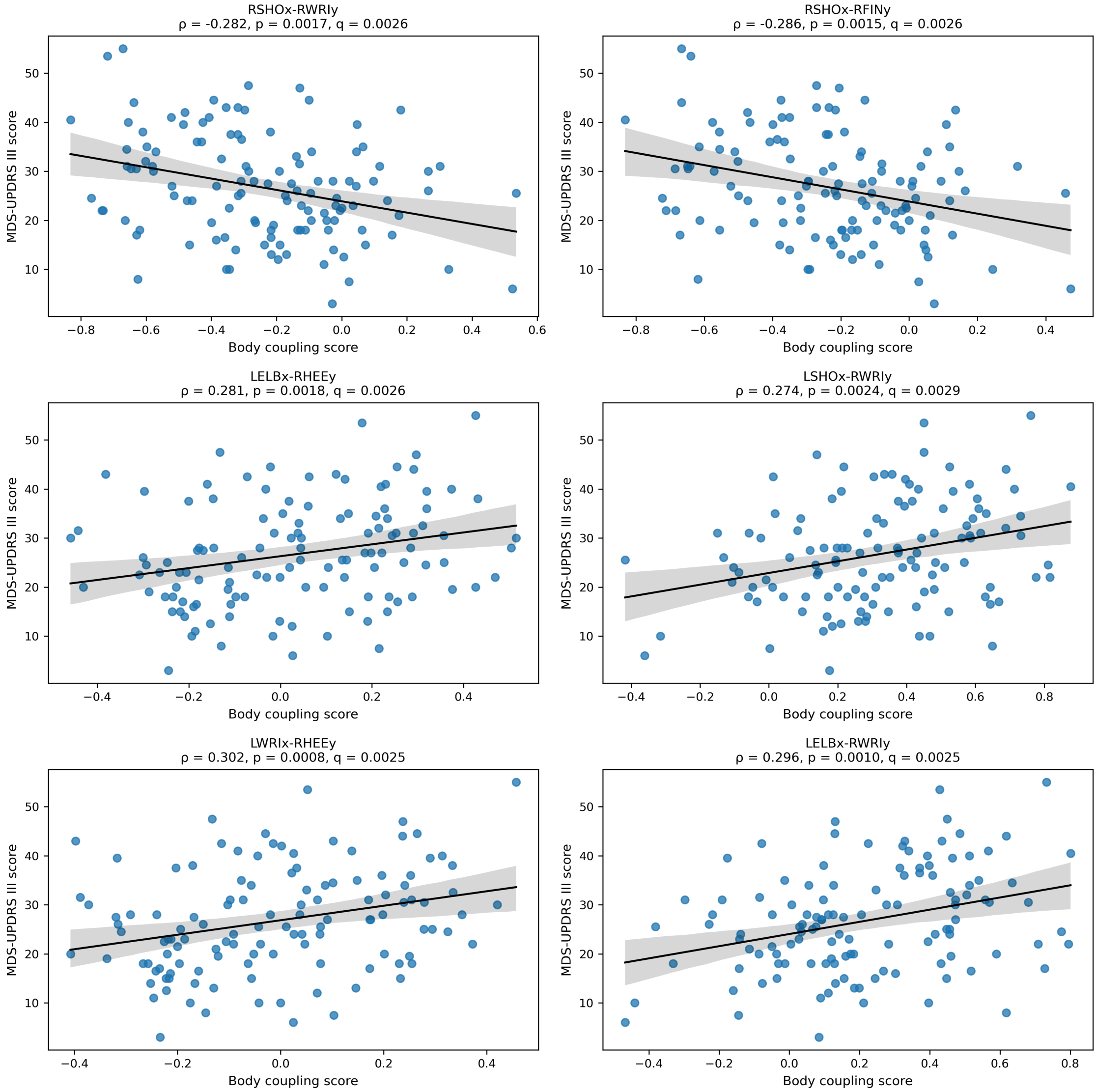


Figure S3: Continued

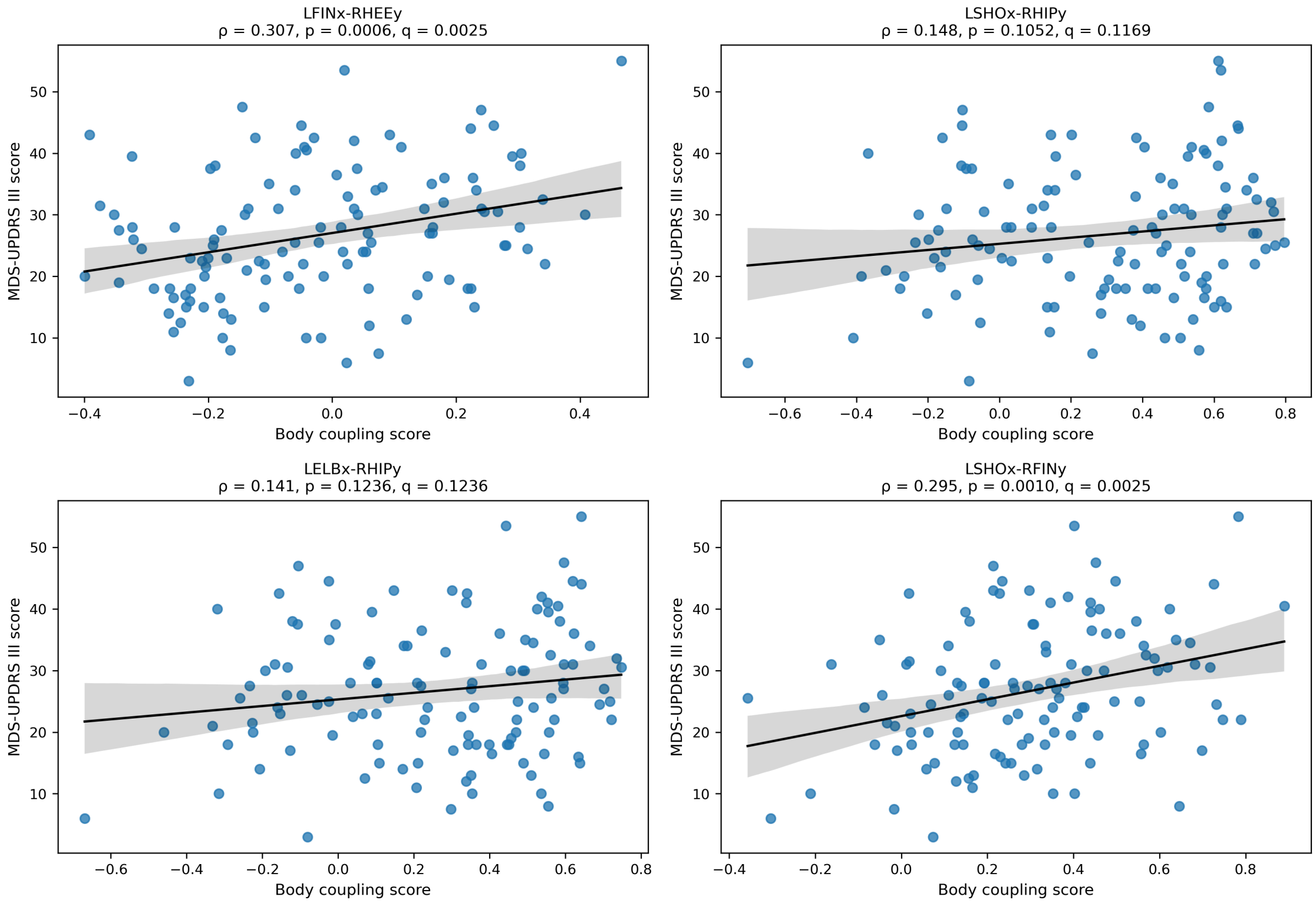


Figure S3: **Spearman correlations between the top 10 directional body coupling features and MDS-UPDRS III scores in the full PD cohort ($n = 121$).** Each panel shows individual subject-level data points, a fitted regression line, and 95% confidence interval. Spearman $\rho$, nominal $p$-value, and FDR-corrected $q$-value are reported in each panel title. Significant associations are shown for RSHOx–RWRIy, RSHOx–RFINy, LELBx–RHEEy, LSHOx–RWRIy, LWRIx–RHEEy, LELBx–RWRIy, LFINx–RHEEy, and LSHOx–RFINy, whereas LSHOx–RHIPy and LELBx–RHIPy did not survive FDR correction.

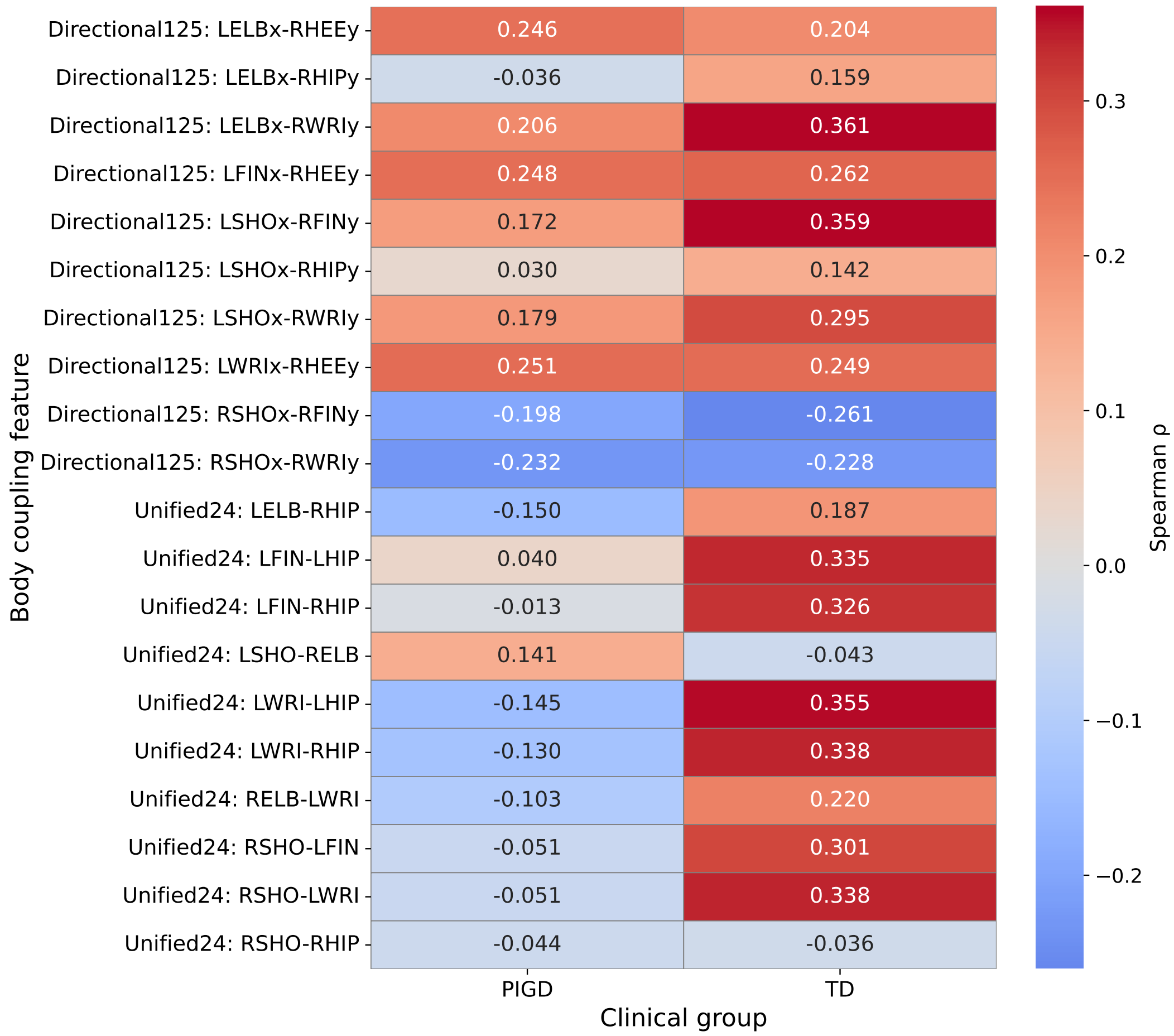


Figure S4: **Exploratory subtype-stratified heatmap of Spearman correlations between top 10 directional and unified body coupling features and MDS-UPDRS III scores in the PIGD ($n$ = 53) and TD ($n$ = 49) subgroups.** Rows show the top 10 directional body coupling features and the top 10 unified body coupling features. Columns represent the PIGD and TD subgroups. Color indicates Spearman $\rho$, with warmer colors representing positive correlations and cooler colors representing negative correlations. These subtype-stratified results are exploratory and should be interpreted cautiously because subgroup sizes were limited and imbalanced.

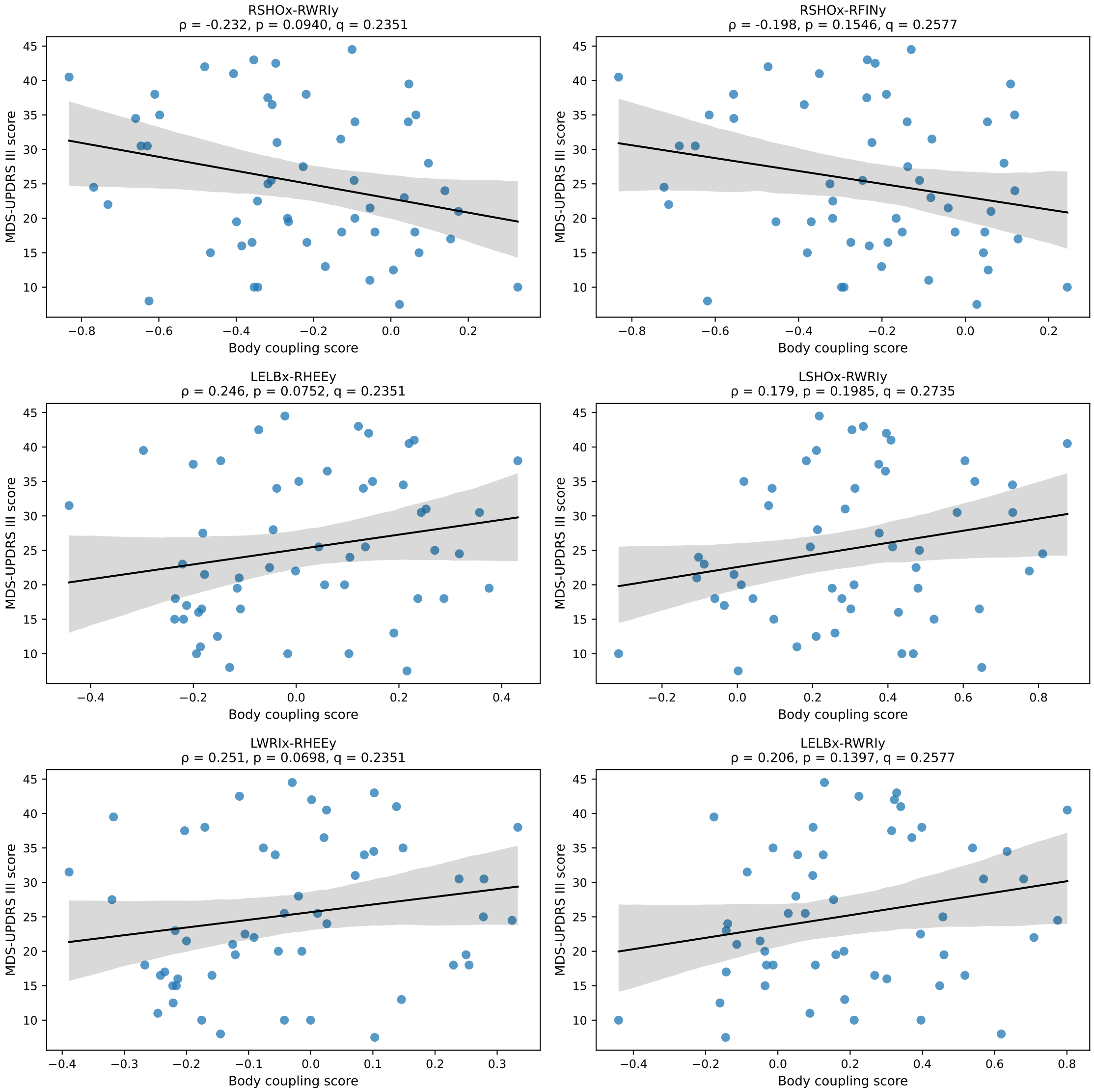


Figure S5: Continued

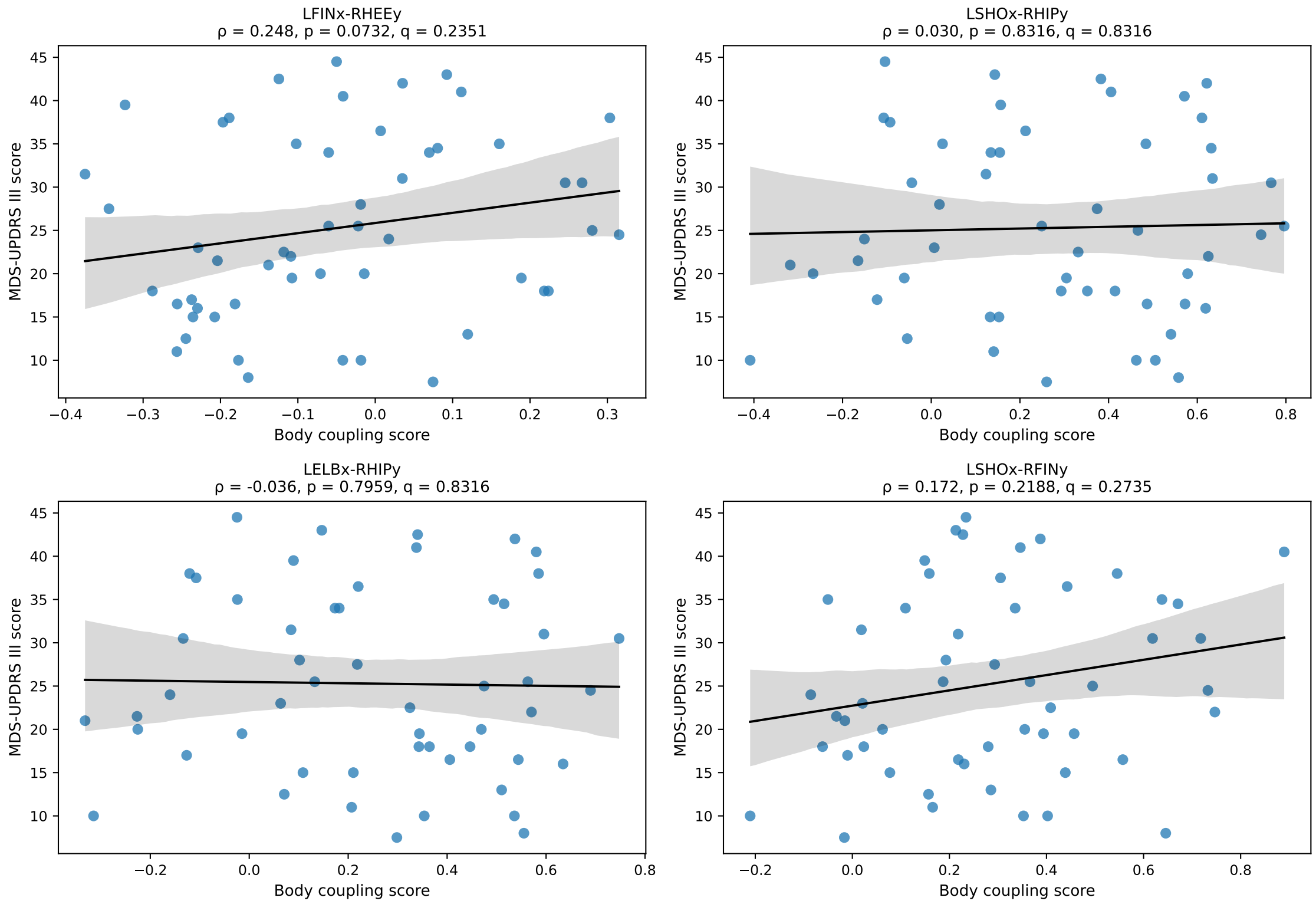


Figure S5: **Exploratory Spearman correlations between the top 10 directional body coupling features and MDS-UPDRS III scores within the PIGD subgroup ($n = 53$).** Each panel shows individual subject-level data points, a fitted regression line, and 95% confidence interval. Spearman $\rho$, nominal $p$-value, and FDR-corrected $q$-value are reported in each panel title. These subtype-stratified results are exploratory and require confirmation in larger, balanced subtype cohorts.

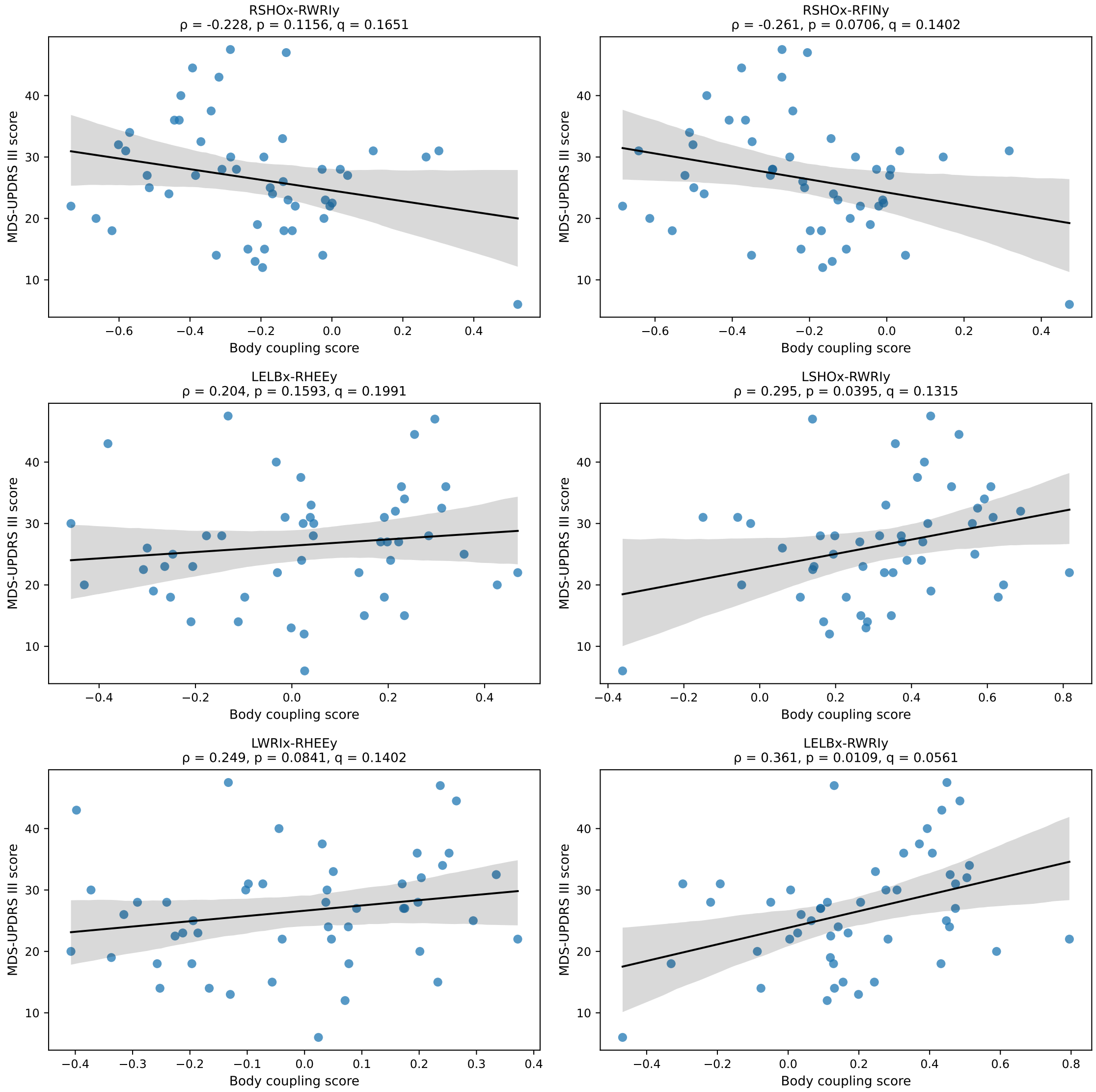


Figure S6: Continued

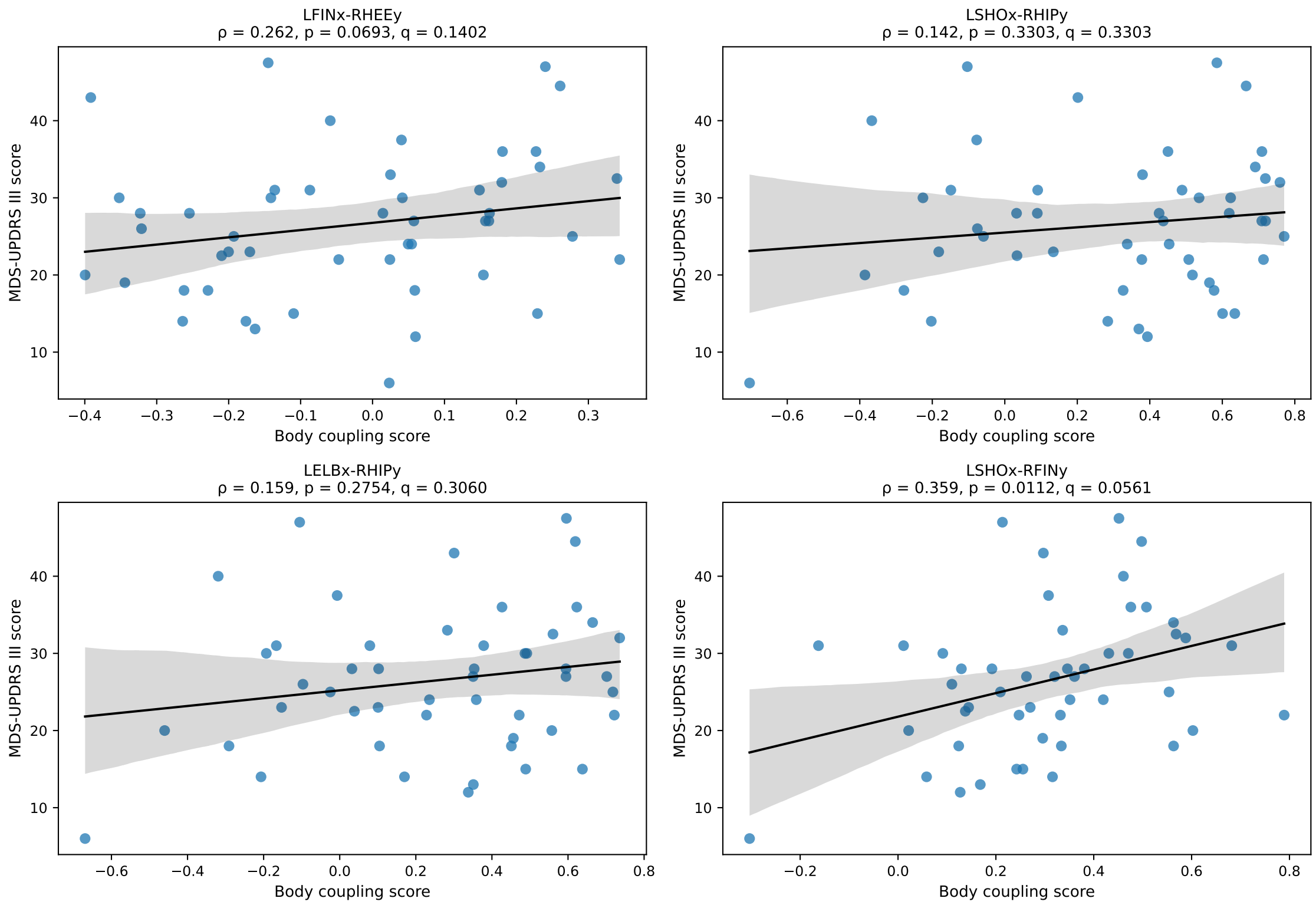


Figure S6: **Exploratory Spearman correlations between the top 10 directional body coupling features and MDS-UPDRS III scores within the TD subgroup ($n = 49$).** Each panel shows individual subject-level data points, a fitted regression line, and 95% confidence interval. Spearman $\rho$, nominal $p$-value, and FDR-corrected $q$-value are reported in each panel title. None of these features survived FDR correction at $q < 0.05$, and these results require validation in a larger TD cohort. Figure continued on next page.

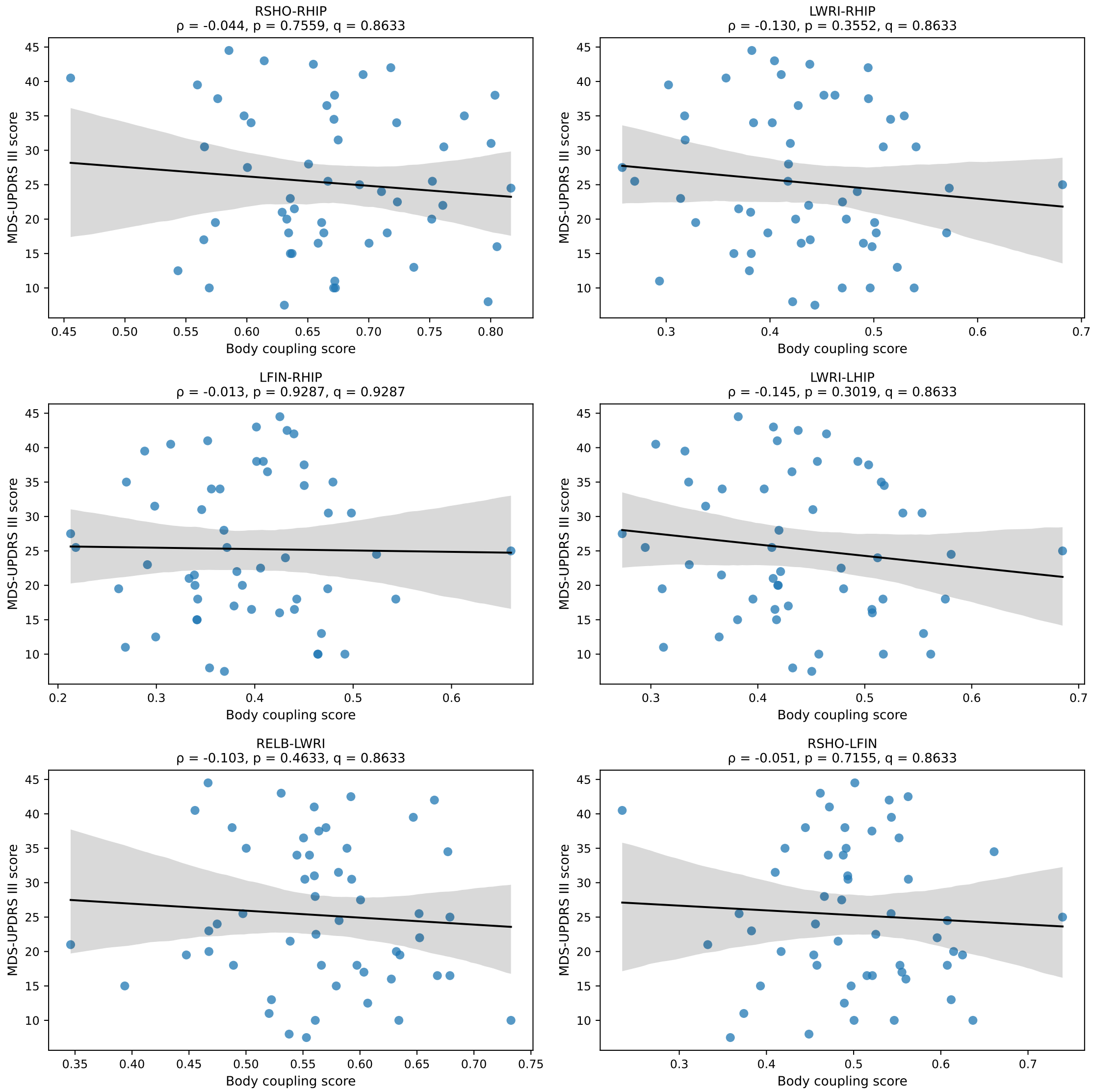


Figure S7: Continued

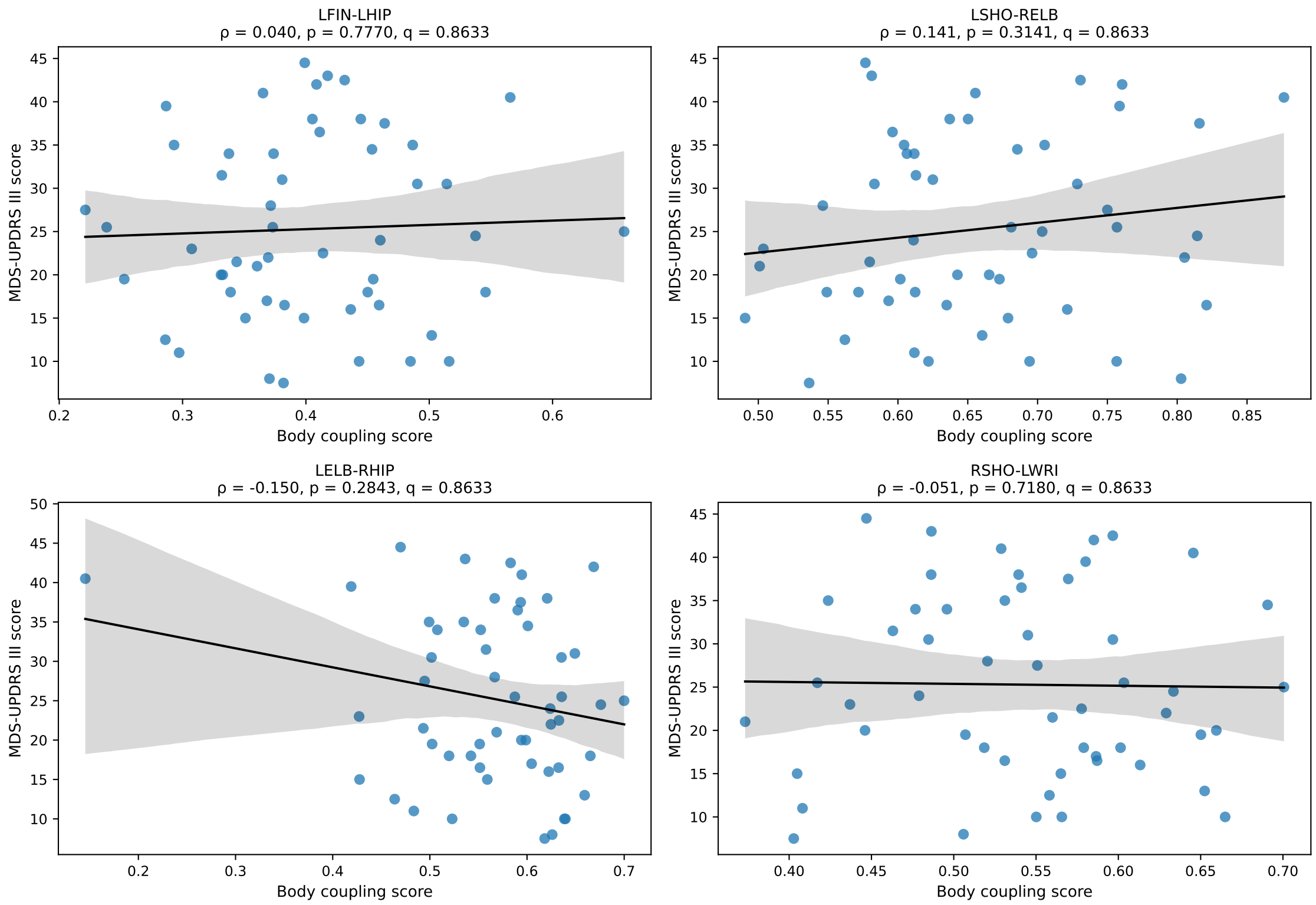


Figure S7: **Exploratory Spearman correlations between the top 10 unified body coupling features and MDS-UPDRS III scores within the PIGD subgroup ($n = 53$).** Each panel shows individual subject-level data points, a fitted regression line, and 95% confidence interval. Spearman $\rho$, nominal $p$-value, and FDR-corrected $q$-value are reported in each panel title. These associations were weak and inconsistent in the PIGD subgroup and should be interpreted cautiously.

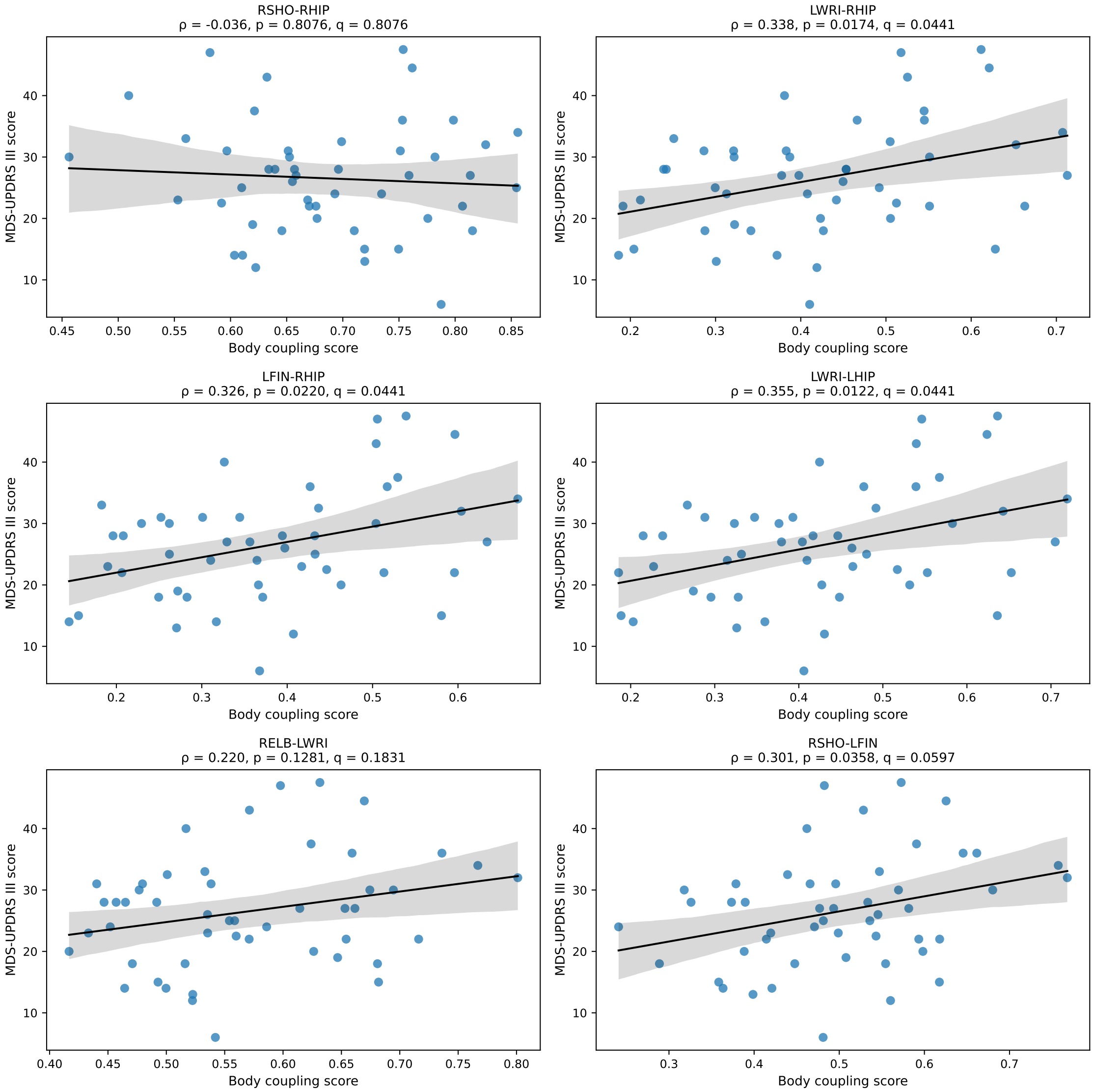


Figure S8: Continued

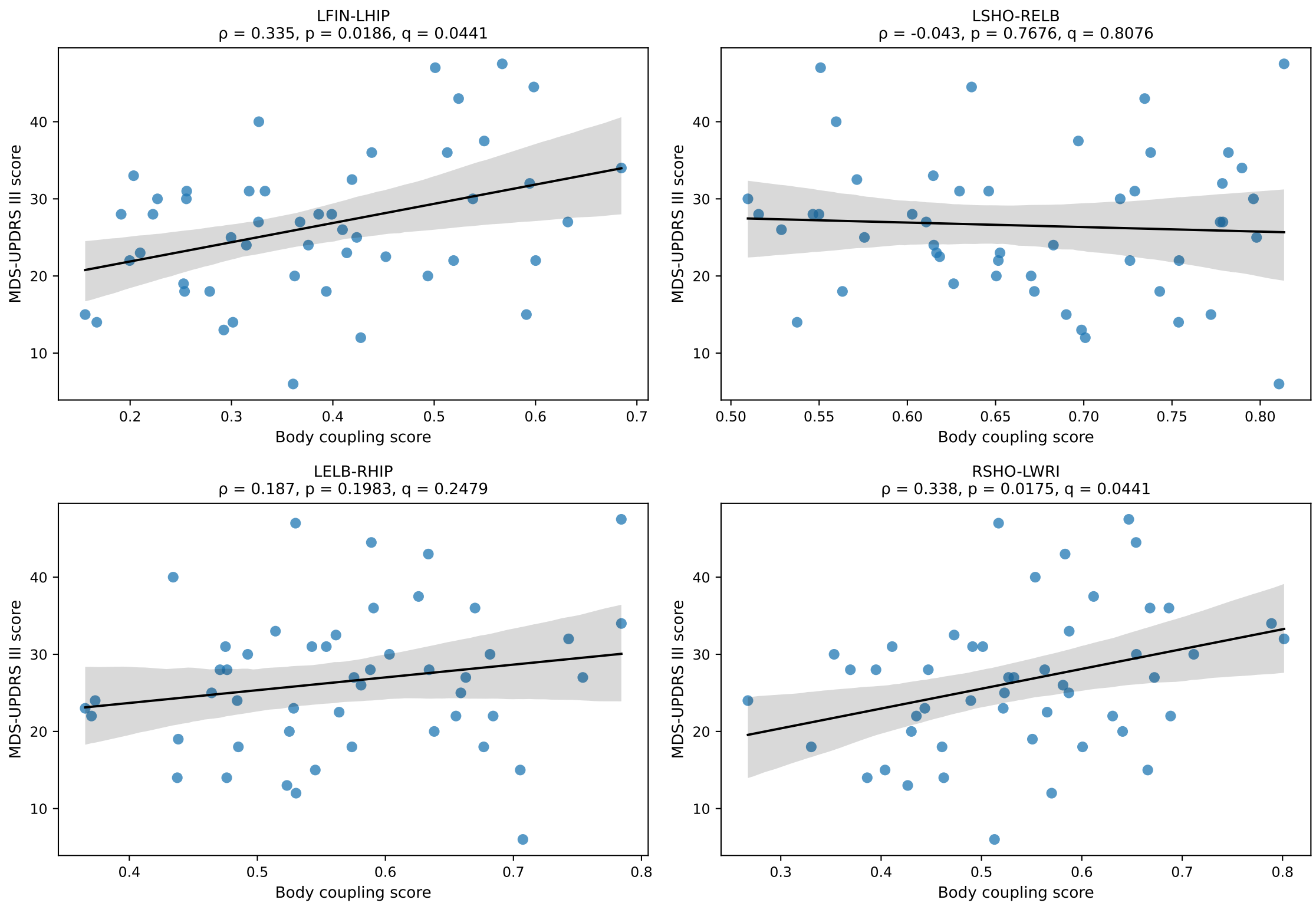


Figure S8: **Exploratory Spearman correlations between the top 10 unified body coupling features and MDS-UPDRS III scores within the TD subgroup ($n = 49$).** Each panel shows individual subject-level data points, a fitted regression line, and 95% confidence interval. Spearman $\rho$, nominal $p$-value, and FDR-corrected $q$-value are reported in each panel title. Several of these features showed positive associations after FDR correction, but these findings remain exploratory because of limited subtype sample size and require independent validation in larger, balanced subtype cohorts.

# References

[1] Glenn T. Stebbins, Christopher G. Goetz, David J. Burn, Joseph Jankovic, Teng L. Khoo, and Barbara C. Tilley. How to identify tremor dominant and postural instability/gait difficulty groups with the movement disorder society unified parkinson's disease rating scale: comparison with the unified parkinson's disease rating scale. *Movement Disorders*, 28(5):668–670, 2013.
